# PIEZO1 mediates periostin^+^ myofibroblast activation and pulmonary fibrosis in mice

**DOI:** 10.1172/JCI184158

**Published:** 2025-06-02

**Authors:** Liran Xu, Ting Li, Yapeng Cao, Yu He, Zehua Shao, Siyu Liu, Bianbian Wang, Ailing Su, Huijing Tian, Yongxin Li, Guozheng Liang, Changhe Wang, John Shyy, Ying Xiong, Fangyuan Chen, Jason X.J. Yuan, Junjun Liu, Bin Zhou, Nina Wettschureck, Stefan Offermanns, Yang Yan, Zuyi Yuan, Shengpeng Wang

**Affiliations:** 1 Department of Cardiac Surgery, the First Affiliated Hospital of Xi’an Jiaotong University, Key Laboratory of Environment and Genes Related to Diseases, Ministry of Education,; 2 Center for Mitochondrial Biology and Medicine, School of Life Science and Technology, and; 3 Biobank, The First Affiliated Hospital of Xi’an Jiaotong University, Xi’an, Shaanxi, China.; 4 Department of Pharmacology, Max Planck Institute for Heart and Lung Research, Bad Nauheim, Germany.; 5 Division of Cardiology, Department of Medicine, University of California, San Diego, La Jolla, California, USA.; 6 Department of Cardiovascular Medicine, The First Affiliated Hospital of Xi’an Jiaotong University, Xi’an, Shaanxi, China.; 7 The Herbert Wertheim UF Scripps Institute, University of Florida, Jupiter, Florida, USA.; 8 Shaanxi Techshake Biotechnology, Xi’an, Shaanxi, China.; 9 New Cornerstone Science Laboratory, State Key Laboratory of Cell Biology, Shanghai Institute of Biochemistry and Cell Biology, Center for Excellence in Molecular Cell Science, Chinese Academy of Sciences, University of Chinese Academy of Sciences, Shanghai, China.

**Keywords:** Cell biology, Pulmonology, Fibrosis, Ion channels

## Abstract

Idiopathic pulmonary fibrosis (IPF) is a devastating interstitial lung disease characterized by the excessive accumulation of activated myofibroblasts that deposit extracellular matrix (ECM) protein, leading to progressive scar formation and mechanical stress. However, the cellular origin and fate of myofibroblasts remain controversial, and the mechanisms by which myofibroblasts sense mechanical cues in the lung are unclear. Here, we report that periostin (Postn) is a reliable and distinctive marker for pulmonary myofibroblasts, while ablation of Postn^+^ myofibroblasts after injury ameliorated lung fibrosis. PIEZO1 was highly expressed in Postn^+^ myofibroblast and played a vital role in mechanoactivation of Postn^+^ myofibroblast and development of lung fibrosis. Conditional deletion of *Piezo1* in Postn^+^ myofibroblasts significantly inhibited lung fibrosis by suppressing myofibroblast activation and proliferation. Loss of *Piezo1* led to disruption of actin organization and prevention of Yap/Taz nuclear localization, thus shifting the myofibroblasts from a proliferative state into a stressed and apoptotic state. Furthermore, myofibroblast-specific *Yap/Taz* deletion fully recapitulated the protective phenotypes of myofibroblast-*Piezo1*–KO mice. These findings show that periostin marks pulmonary myofibroblasts, and that PIEZO1-mediated mechanosensation is essential for myofibroblast activation in the lung. Targeting PIEZO1 in the periostin-expressing cells is a novel therapeutic option to interfere with fibrotic diseases such as IPF .

## Abstract 2

PIEZO1 plays a vital role in the mechano-activation of Postn^+^ myofibroblast during the development of lung fibrosis.

## Graphical abstract

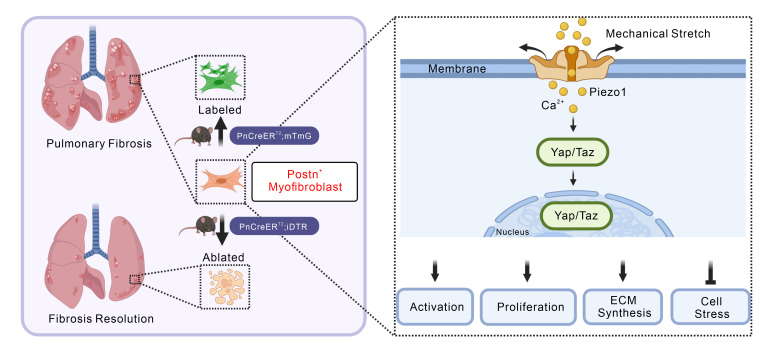

## Introduction

Idiopathic pulmonary fibrosis (IPF) is a devastating interstitial lung disease with poor prognosis of less than 5 years after diagnosis (1, 2). To date, highly effective treatments for IPF are lacking, mostly due to the incomplete understanding of its pathogenesis (3–5). The hallmark feature of lung tissues from patients with IPF is the excessive accumulation of activated myofibroblasts that deposit extracellular matrix (ECM) protein, which leads to progressive scar formation and increasing stiffness, thus ultimately causing irreversible obstruction of airways (3–5). However, the cellular origin and fate of myofibroblasts in pulmonary fibrosis have remained controversial (5–8).

Multiple cell populations have been proposed as the precursors of activated myofibroblasts in IPF (7). Of these, quiescent resident fibroblasts represent important precursors of myofibroblasts (8, 9). Tsukui et al. recently showed that lung-specific alveolar Scube2^+^ fibroblasts are the source of both inflammatory and fibrotic fibroblasts (10). Fate mapping studies implied that lung myofibroblasts originate from bone marrow-derived fibrocytes and epithelial cells undergoing epithelial-to-mesenchymal transition (EMT), whereas another study indicates that circulating fibrocytes and epithelial cells, including type 2 alveolar epithelial cells (AEC2), are not major sources of myofibroblasts (11, 12). Pericytes were proposed as another main source of the myofibroblast pool in the lung; however, this hypothesis remains controversial (6, 12). A recent study reported that Sfrp1^+^ transitional fibroblasts gave rise to myofibroblasts after lung injury (13). The other debated sources of myofibroblasts include ADRP^+^ lipofibroblasts (14), Axin2^+^ progenitors (15), Gli1^+^, and PDGFR-β^+^ mesenchymal cells (16, 17).

Beyond cellular origin, the lack of selective myofibroblast markers also hampers the identification and targeting of these cells in the injured lung (7, 18). α-SMA was widely accepted as a pulmonary myofibroblast marker, but it is constitutively expressed in vascular smooth muscle cells (VSMCs). Classical fibroblast makers, including platelet-derived growth factor receptor-α (PDGFR-α), fibroblast-specific protein–1 (FSP1), collagen, or vimentin appear to identify myofibroblasts, unfortunately, none of them were exclusive. Recent studies showed that PDGFR-β marks most myofibroblasts in the injured lung (17), but PDGFR-β is expressed in diverse stromal and perivascular cells. In addition, due to the lack of a myofibroblast-specific lineage tracing tools, the cell fate of pulmonary myofibroblasts is often inaccurately or contradictorily interpreted. We recently developed a myofibroblast-specific transgenic mouse line allowing tamoxifen-inducible Cre expression in periostin-positive cells (*Postn-CreER^T2^*) (19). Periostin (Postn) is an ECM protein that binds to collagen and fibronectin. It is expressed in myofibroblasts and promotes fibrosis in response to tissue injury (19–21). Fate mapping with *Postn-CreER^T2^* mice suggests that Postn^+^ cells mark almost all myofibroblasts after injury of heart and liver, and ablation of Postn^+^ cells ameliorates fibrosis (19, 22). However, the fate and function of Postn^+^ cells in pulmonary fibrosis remains largely unknown (7, 18).

Lung tissue is constantly exposed to hemodynamic and respiratory mechanical forces during each breathing cycle, and mechanotransduction plays a fundamental role in lung fibrosis (23, 24). Under continued mechanical stress in the fibrotic scar tissue, myofibroblasts fully activated to resemble stress fibers and express α-SMA and other ECM proteins, which promote a positive mechanical feedback loop that amplifies fibrosis (25, 26). In this study, we focus on PIEZO1, a mechanosensitive cation channel that mediates multiple cellular processes, including endothelial flow sensing (27, 28), erythrocyte osmotic regulation (29), and adipocyte (30) as well as cardiomyocyte hypertrophy (31). Recent studies indicate that PIEZO1 is involved in lung innate immunity (32), and that its expression is elevated in vascular cells from patients with pulmonary hypertension (33, 34). However, it is not known whether PIEZO1 is expressed and functional in myofibroblasts in lung fibrosis.

Herein, we demonstrate that Postn is a reliable and distinctive marker for myofibroblasts in the injured lung. By using *Postn-CreER^T2^* mice, we show that Postn identifies myofibroblasts in bleomycin-induced lung fibrosis. Ablation of Postn^+^ myofibroblasts after injury strongly improved lung fibrosis. Furthermore, PIEZO1-mediated mechanosensation is essential for myofibroblast activation in the lung. Conditional *Piezo1* deletion or pharmacological PIEZO1 inhibition in myofibroblasts dramatically improved lung fibrosis. Mechanistically, loss of *Piezo1* led to the disruption of actin organization, prevention of Yap/Taz nuclear localization, and cell stress. Indeed, myofibroblast-specific *Yap/Taz* double deletion fully recapitulated the protective phenotypes of myofibroblast-*Piezo1*–KO mice in the bleomycin injury model. Thus, Postn labels pulmonary myofibroblasts and PIEZO1 plays a vital role in the mechano-activation of Postn^+^ myofibroblasts during the development of lung fibrosis.

## Results

### Postn predominantly labels myofibroblasts in lung injury.

**Figure 1:**
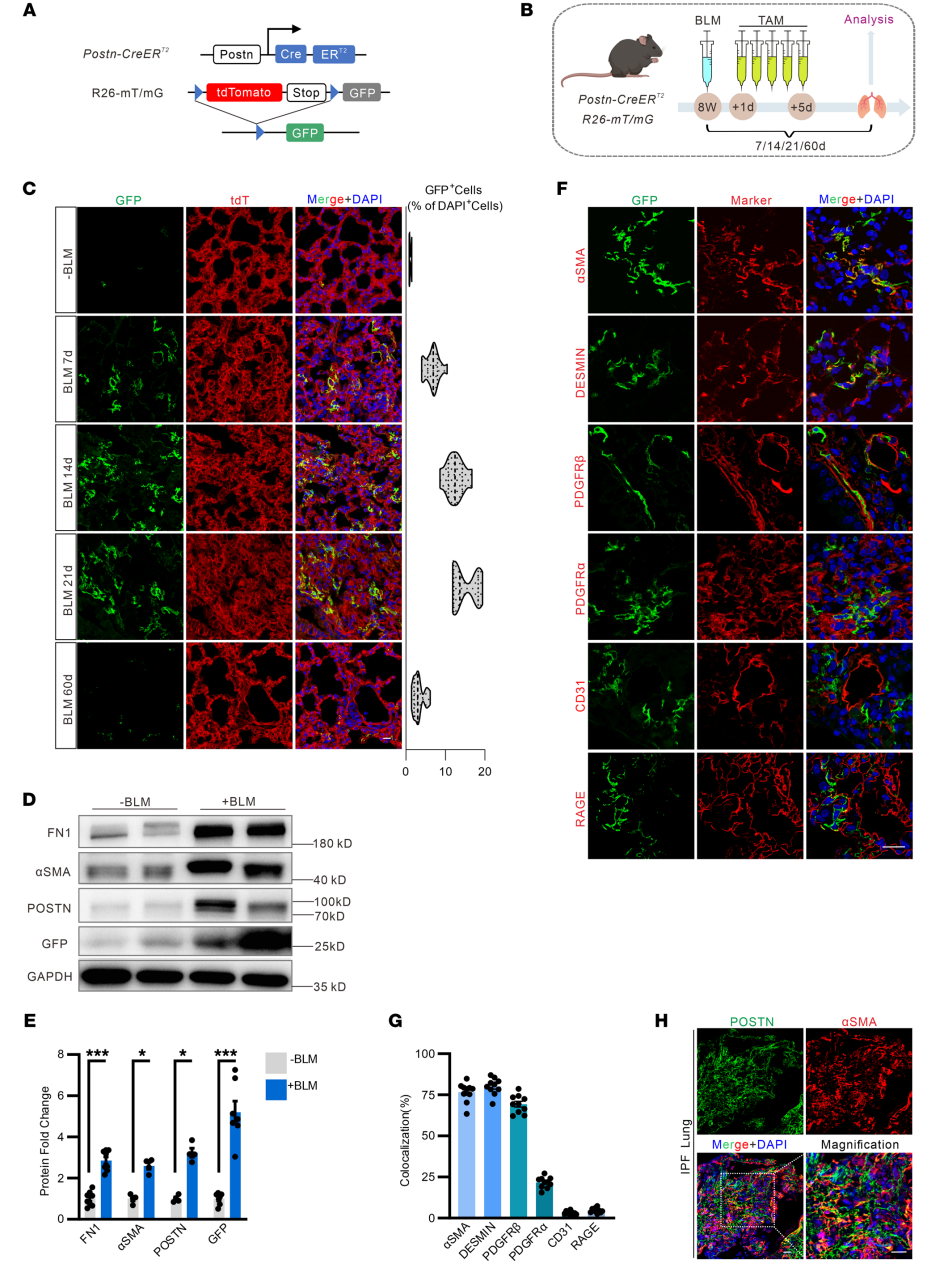
Postn^+^ cell lineage tracing during lung fibrosis. (**A**) Schematic representation showing the genetic strategy for the generation of the *Postn-CreER^T2^; mT/mG* mice for lineage tracing. (**B**) Schematic diagram of the experimental design. *Postn-CreER^T2^; mT/mG* mice were challenged to a single intratracheal inhalation of 1 U/kg BLM followed by injection with tamoxifen on 5 consecutive days. The control mice (*Postn-CreER^T2^; mT/mG*) without BLM injury (–BLM) were induced by 5 consecutive days tamoxifen and analyzed at day 21 after tamoxifen injection. (**C**) Representative images of Postn^+^ lineage GFP^+^ cells in *Postn-CreER^T2^; mT/mG* mice after tamoxifen treatment and BLM challenge. Immunofluorescent staining showing tdTomato and GFP single channels in addition to a merged image. Scale bar: 20 μm. (**D** and **E**) Western blot analysis (**D**) and quantification (**E**) of the indicated protein levels in the lung of mice at 21 days post bleomycin injury (d.p.i.). *n* = 4–8. (**F**) Representative images to identify the Postn^+^ cells in the mouse lung sections. Antibodies against the stromal markers (α-SMA, Desmin, Pdgfr-β, and Pdgfr-α), endothelial marker CD31, and alveolar type 1 (AT1) cell marker Rage were costained in the lung sections of *Postn-CreER^T2^; mT/mG* mice at 21 d.p.i. Scale bar: 20 μm. (**G**) Quantification of colocalization of the GFP^+^ cells with α-SMA^+^, Desmin^+^, Pdgfr-β^+^, Pdgfr-α^+^, CD31^+^, or Rage^+^ cells in the lung sections of *Postn-CreER^T2^; mT/mG* mice at 21 d.p.i. *n* = 10. (**H**) Representative images of Postn expression, which were costained with α-SMA in the lung sections of patients with IPF. Scale bar: 20 μm. *n* = 8. Shown are mean values ± SEM. Statistical significance was determined by unpaired Student’s *t* test or the Mann-Whitney *U* test. ***P < 0.001.

To genetically label Postn-expressing cells in vivo, we crossed *Postn-CreER^T2^* with the *mT/mG* reporter line, which switches from membrane-targeted Tomato expression to membrane-targeted *GFP* expression upon Cre-mediated recombination (Figure 1, A and B). In healthy adult tissues, including the lung, Postn^+^ GFP cells are rarely detected at basal conditions (Figure 1C). Also, rare GFP cells could be detected when tamoxifen-induced Postn^+^ cell labeling was performed prior to bleomycin injury (Supplemental Figure 1, A and B; supplemental material available online with this article; https://doi.org/10.1172/JCI184158DS1). To initially assess the expression of Postn upon lung injury, *Postn-CreER^T2^; mT/mG* mice were subjected to a single bleomycin instillation and then 5 days of tamoxifen labeling (Figure 1B). At 7 days post injury (d.p.i.), a few Postn^+^ GFP cells were present in lung tissues and the number of Postn^+^ cells increased significantly at 14 and peaked at 21 d.p.i. (Figure 1C). The GFP-labeled cells were dramatically decreased at 60 d.p.i., when the lung fibrosis was nearly resolved. At 21 d.p.i., abundant expression of Postn, GFP, as well as α-SMA and fibronectin 1 (Fn1) were detected in the lung from bleomycin-treated *Postn-CreER^T2^;mT/mG* mice, while PBS-treated mice had almost no GFP and Postn in the lung (Figure 1, D and E). Then, the identity of Postn^+^ cells was interrogated with a combination of markers for stromal, endothelial, and epithelial cells. After lung injury, quantification of the immunofluorescent staining showed that the majority of Postn^+^ cells were positive for conventional myofibroblast makers, including α-SMA, Desmin, and Pdgfr-β, while about 20% were Pdgfr-α (fibroblast marker) positive but few were CD31 (endothelial marker) or Rage (alveolar epithelial maker) positive (Figure 1, F and G). In myofibroblasts differentiated from human lung fibroblast MRC5 by 72 hour TGF-β pretreatment, siRNA-mediated Postn knockdown led to a strong reduction in cell contraction and actin stress fibers (Supplemental Figure 1, C and D). Finally, in lung tissue samples from patients with IPF, abundant Postn^+^ cells were present in the fibroblastic foci (Figure 1H). Further analysis of single cell transcriptomics (GSE136831 and GSE129605) reveals the distinctive Postn expression in myofibroblasts from diseased lung (Supplemental Figure 1, E–H). Thus, our results indicate that Postn is rarely present in the healthy state but marks myofibroblasts of the fibrotic lung.

### Ablation of Postn^+^ cells mitigates pulmonary fibrosis.

**Figure 2:**
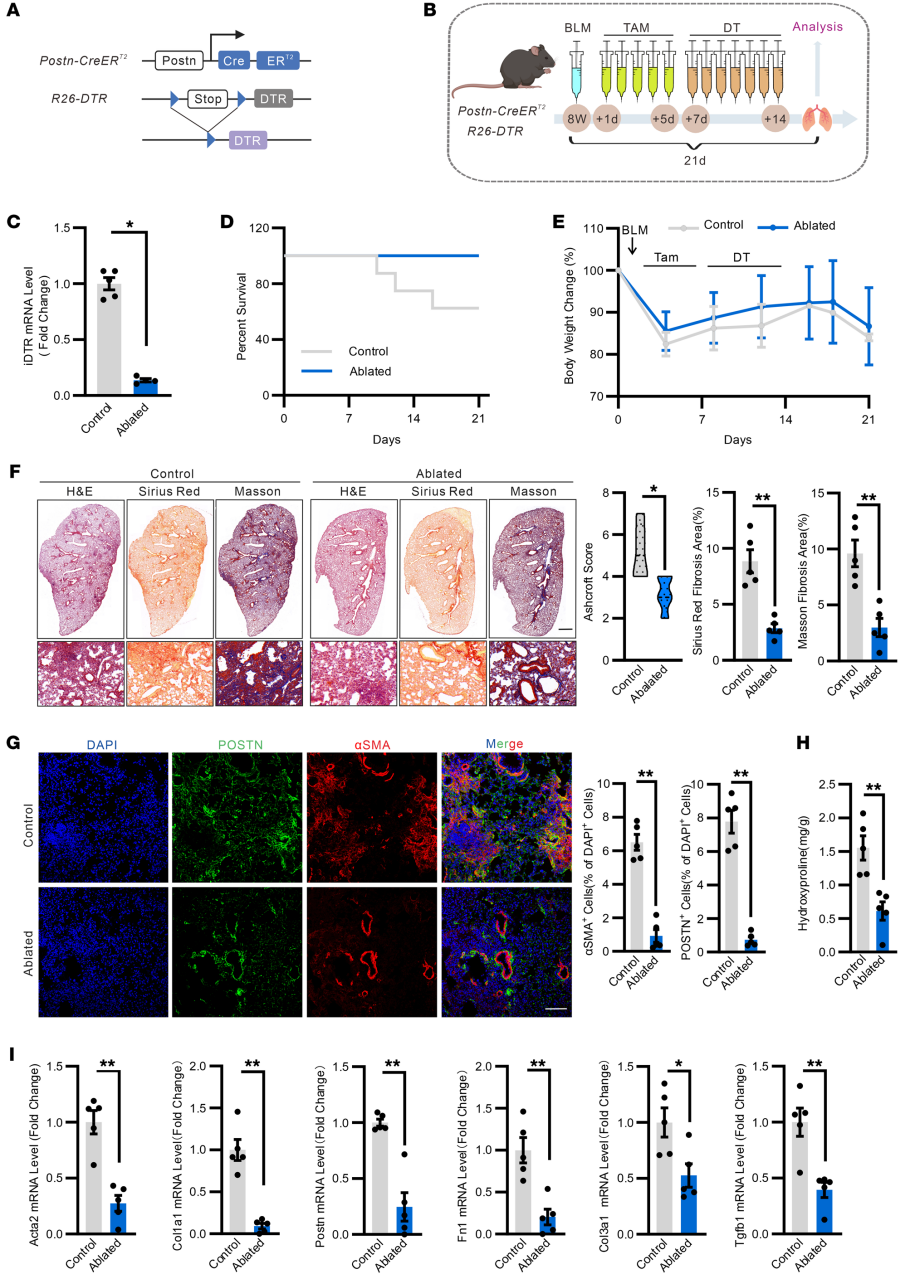
Postn^+^ cell ablation mitigates BLM-induced pulmonary fibrosis in mice. (**A**) Schematic representation showing the genetic strategy for generation of the *Postn-CreER^T2^; R26-iDTR* mice for ablation of Postn^+^ cells. (**B**) Schematic diagram of the experimental design. *Postn-CreER^T2^; R26-iDTR* were challenged to a single intratracheal inhalation of BLM followed by injection with tamoxifen on 5 consecutive days. Then the mice were injected with DT or vehicle for 7 consecutive days. After 7 days, the control (*R26-iDTR*) and *Postn-CreER^T2^; R26-iDTR* mice were euthanized for subsequent analysis. (**C**) Real-time qPCR analysis of *iDTR* mRNA expression levels in the lung of *Postn-CreER^T2^; R26-iDTR* and control mice. *n* = 4–5. (**D** and **E**) Survival percentage (**D**) and body weight change (**E**) of *Postn-CreER^T2^; R26-iDTR* and control mice. *n* = 5. (**F**) (*left*) Representative images of H&E, Sirius Red, and Masson staining in the lung sections of *Postn-CreER^T2^; R26-iDTR* mice after Postn^+^ cell ablation. (*right*) Quantification of the Ashcroft score, Sirius red fibrosis and Masson fibrosis area. (Scale bar: 1 mm, top; 100 μm, bottom). *n* = 5. (**G**) Representative images of Postn and α-SMA expression and quantification of α-SMA^+^ and Postn^+^ area in the lung sections of *Postn-CreER^T2^; R26-iDTR* mice after Postn^+^ cells ablation. Scale bar:100 μm. *n* = 5. (**H**) Hydroxyproline content in the lung of ablated and control mice after BLM challenge. *n* = 5. (**I**) Real-time qPCR analysis of *Acta2, Col1a1, Postn, Fn1, Col3a1,* and *Tgfb1* mRNA expression levels in the lungs of *Postn-CreER^T2^; R26-iDTR* mice after the Postn^+^ cells ablation. *n* = 5. Shown are mean values ± SEM. Statistical significance was determined by the Mann-Whitney *U* test. **P < 0.01.

To assess the relevant contribution of Postn^+^ cells to lung fibrosis, we crossed *Postn-CreER^T2^* mice to the inducible human diphtheria toxin receptor (iDTR) mouse line (35). *iDTR* mice express the DTR upon Cre-mediated recombination, rendering Cre-expressing cells sensitive to diphtheria toxin (Figure 2A). To selectively ablate Postn^+^ cells, *Postn-CreER^T2^; iDTR* bigenic mice were first treated with a single intratracheal inhalation of bleomycin, and Cre recombination was induced by 5 consecutive days of tamoxifen injection followed by 7 days of diphtheria toxin daily treatment (Figure 2B). Successful ablation of Postn^+^ cells was verified by quantitative PCR for DTR expression (Figure 2C). Notably, ablation of Postn^+^ cells improved the survival rate and the body weight loss after injury (Figure 2, D and E). More important, there was also a remarkable reduction in lung fibrosis after Postn^+^ cell ablation (Figure 2F). Histology and fluorescent staining in ablated lungs showed that not only a predominant reduction of Postn expression but also a generally abolished profibrotic activity (Figure 2, F and G). Furthermore, ablation of Postn^+^ cells strongly suppressed injury-induced hydroxyproline content and expression of key fibrotic genes, including *Acta2*, *Col1a1, Postn, Fn1, Col3a1,* and *Tgfb1* (Figure 2, H and I). These data provide strong evidence that Postn^+^ cells are essential for the formation of lung fibrosis after injury.

### Functional expression of PIEZO1 in lung myofibroblasts.

**Figure 3:**
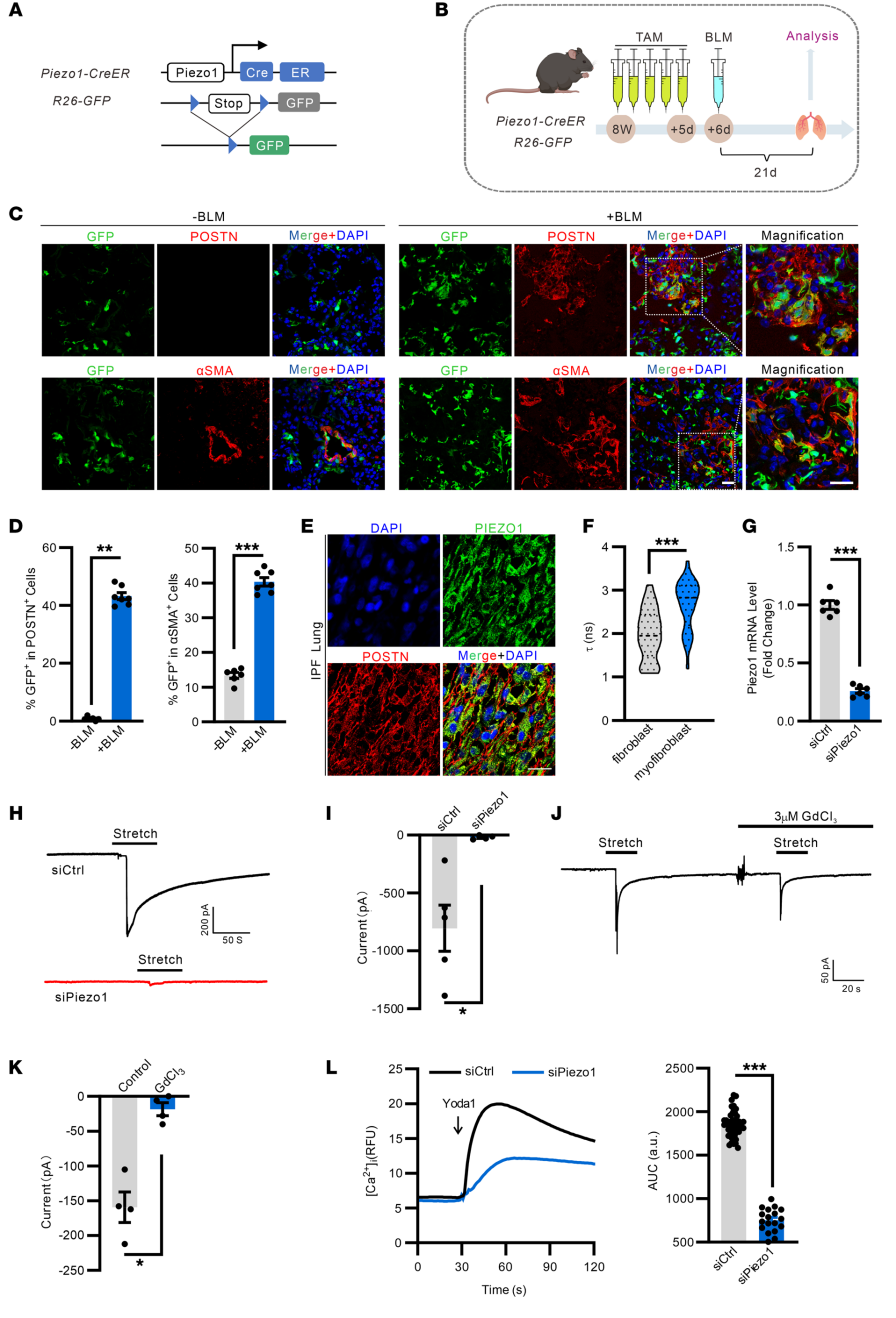
PIEZO1^+^ cell tracing and functional characterization of PIEZO1 in myofibroblasts. (**A**) Schematic representation showing the genetic strategy for the generation of the *Piezo1-CreER; R26-GFP* mice. (**B**) Schematic diagram of the experimental design. *Piezo1-CreER; R26-GFP* mice were injected with tamoxifen for 5 consecutive days followed by a single intratracheal inhalation of bleomycin (+BLM) or vehicle (–BLM). (**C**) Representative images of PIEZO1 expression in the Postn^+^ or α-SMA^+^ area before and after BLM challenge of *Piezo1-CreER;R26-GFP* mice, respectively. Scale bar: 20 μm. (**D**) Quantification of GFP^+^ in Postn^+^ or α-SMA^+^ cells. *n* = 6–7. (**E**) Representative images of PIEZO1 expression in the lung sections of patients with IPF. Scale bar: 20 μm. (**F**) Quantification of membrane tension before and after myofibroblast differentiation. *n* = 32. (**G**) Relative *Piezo1* mRNA levels in myofibroblasts transfected with siCtrl or with siPiezo1. *n* = 6. (**H**) Representative cell-attached patch clamp traces of stretch-activated currents in myofibroblasts transfected with siCtrl or with siPiezo1. The holding potential was –70 mV and the membrane was stretched by pulses of negative pressure with a 10 mm Hg increment. (**I**) Statistical analysis of 4–5 independent recordings. (**J**) Representative cell-attached patch clamp traces of stretch-activated currents in myofibroblasts exposed to GdCl_3_ or PBS. The holding potential was –70 mV and the membrane was stretched by pulses of negative pressure with a 10 mm Hg increment. (**K**) Statistical analysis of 4 independent recordings. (**L**) Fluo-4–loaded myofibroblasts transfected with siCtrl or with siPiezo1 were exposed to 1 μM Yoda1, and [Ca^2+^]_i_ was determined as fluorescence intensity (RFU, relative fluorescence units); line indicates the addition of Yoda1. Bar diagrams show the AUC of the Ca^2+^ transient. *n* = 18–45. Shown are mean values ± SEM. Statistical significance was determined by unpaired Student’s *t* test or the Mann-Whitney *U* test. **P < 0.01; ***P < 0.001.

Myofibroblast activation is synergistically promoted by biochemical and mechanical stimuli, and disrupted mechanical homeostasis is an essential feature of fibrotic tissue (36). PIEZO1 is a predominant mechanosensor expressed in various tissues, including the lung. Here, by crossing a recently developed *Piezo1*-*CreER* knock-in mouse line (37) with the Rosa26 GFP reporter (38), the expression of PIEZO1 in the fibrotic lung was traced during the course of injury (Figure 3A). We treated 8-week-old *Piezo1-CreER*; *GFP* lineage mice with 5-day tamoxifen to permanently label PIEZO1^+^ cells at baseline, followed by a single intratracheal administration of bleomycin, and, at 21 d.p.i., the lung was harvested to detect PIEZO1-traced GFP^+^ cells (Figure 3B). In the healthy lung, immunostaining analysis showed that PIEZO1-traced GFP^+^ cells were mainly endothelial cells and smooth muscle cells (Figure 3C). Upon injury, the number of PIEZO1-traced GFP^+^ cells was significantly increased across the lung, and GFP signals were observed in vascular cells and some hematopoietic and epithelial cells (Figure 3C and Supplemental Figure 2A). Interestingly, costaining with Postn suggested that most injury-induced Postn^+^ cells were GFP-expressing cells in the fibrotic lung (Figure 3, C and D). There is also a strong overlap of GFP signalling with other conventional myofibroblast markers, including α-SMA, Collagen I, and Pdgfr-α in the fibrotic region (Figure 3, C and D and Supplemental Figure 2A). Reanalysis of single cell transcriptomics (GSE136831) reveals considerable overlapping expression of PIEZO1 in Postn^+^ cells from IPF lung (Supplemental Figure 2, B and C). Consistently, in lung sections from patients with IPF, there is a strong expression of PIEZO1 in Postn^+^ cells (Figure 3E). We next studied the functional relevance of PIEZO1 in myofibroblasts differentiated from mouse NIH3T3 and human MRC5 by TGF-β (Supplemental Figure 2, D and E). During the TGF-β–mediated differentiation of fibroblasts into myofibroblasts, the cell membrane tension is significantly increased (Figure 3F). Cell-attached patch-clamp recording of differentiated myofibroblasts revealed the existence of larger stretch-activated currents; this effect was almost lost after knockdown of *Piezo1* using validated siRNAs (Figure 3, G**–**I and Supplemental Figure 3, A and B). Inhibition of PIEZO1 with gadolinium chloride (GdCl_3_) also largely suppressed stretch-activated currents (Figure 3, J and K). Similarly, Yoda1 induced a significant Ca^2+^ influx in control myofibroblasts, which was strongly inhibited in *Piezo1*-deficient cells (Figure 3L and Supplemental Figure 3, C and D).

### Myofibroblast PIEZO1 deletion ameliorates lung fibrosis.

**Figure 4:**
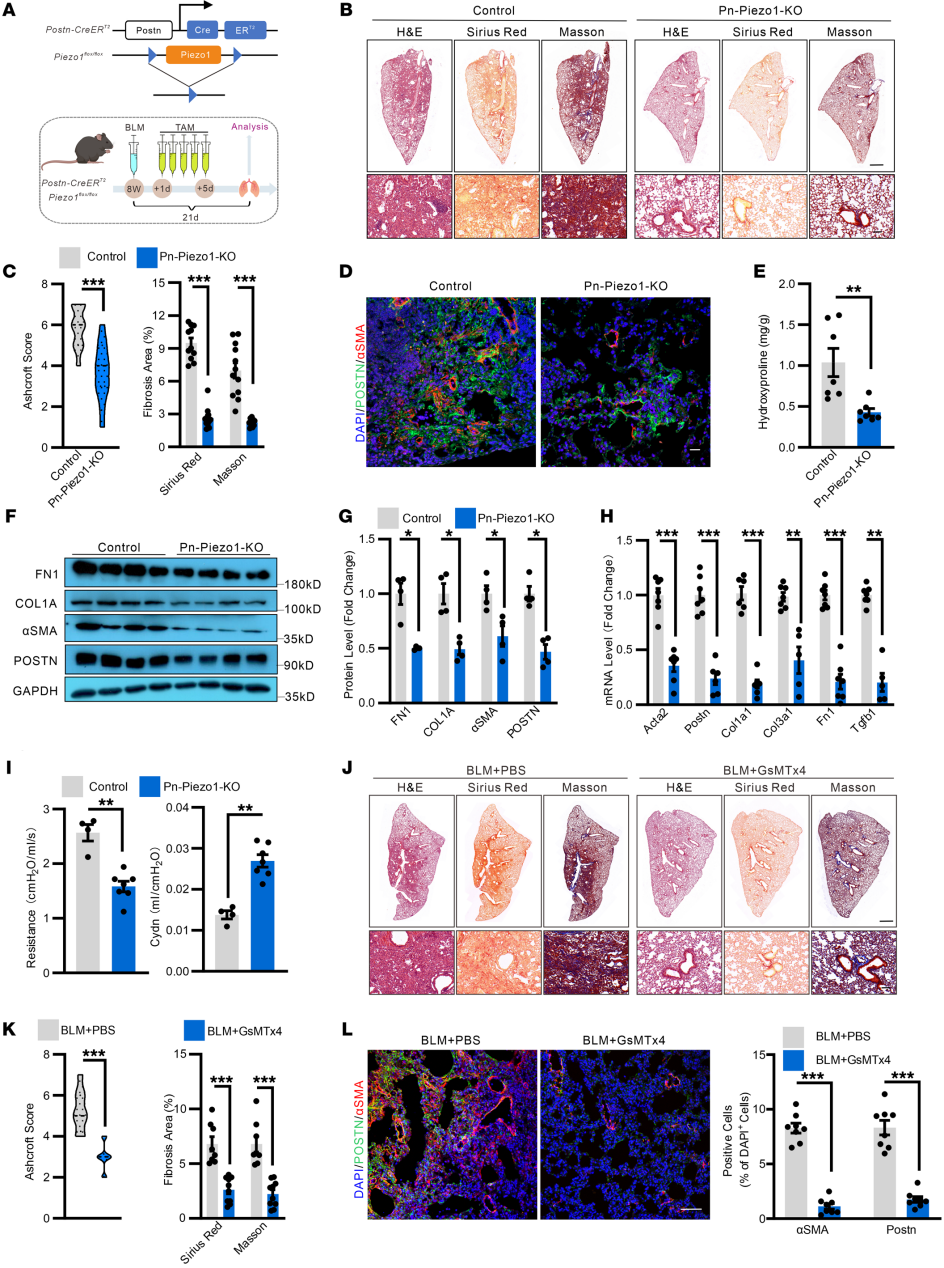
Myofibroblast PIEZO1 promotes bleomycin-induced myofibroblast accumulation and lung fibrosis in mice. (**A**) Schematic representation showing the genetic strategy for the generation of the *Postn-CreER^T2^;Piezo1^fl/fl^* mice (*Pn-Piezo1*-*KO*). And schematic diagram of the experimental design. (**B**) Representative images of H&E, Sirius Red, and Masson staining in the lung sections of *Pn-Piezo1-KO* and control (*Piezo1^fl/fl^*) mice after BLM challenge. (scale bar: 1 mm, top; 100 μm, bottom). (**C**) Quantification of the Ashcroft score, Sirius red, and Masson fibrosis area. *n* = 10–12. (**D**) Representative images of Postn and α-SMA expression in the lung sections of *Pn-Piezo1*–*KO* and control mice after BLM challenge. Scale bar: 20 μm, *n* = 10–12. (**E**) Hydroxyproline content in the lung of *Pn-Piezo1*–*KO* and control mice after BLM challenge. *n* = 7. (**F** and **G**) Western blot analysis (**F**) and quantification (**G**) of Fn1, Col1a1, Acta2, and Postn protein levels in lung homogenates of *Pn-Piezo1*–*KO* and control mice at 21 d.p.i. *n* = 4. (**H**) Relative *Acta2, Postn, Col1a1, Col3a1, Fn1,* and *Tgfb1* mRNA levels in the lung homogenates of *Pn-Piezo1*–*KO* and control mice at 21 d.p.i. *n* = 5–7. (**I**) Lung resistance and dynamic compliance (Cdyn) analysis of *Pn-Piezo1*–*KO* and control mice at 21 d.p.i. *n* = 4–7. (**J**) Representative images of H&E, Sirius Red, and Masson staining in the lung of C57BL/6J mice receiving BLM followed by GsMTx4 treatment. (Scale bar: 1 mm, top; 100 μm, bottom). (**K**) Quantification of the Ashcroft score, Sirius red fibrosis, and Masson fibrosis area. *n* = 8–10. (**L**) (*left*) Representative images of Postn and α-SMA expression in the lung sections of C57BL/6 mice receiving BLM followed by GsMTx4 treatment. Scale bar: 50 μm. (*right*) Quantification of α-SMA^+^ and Postn^+^ area in the lung of C57BL/6J mice receiving BLM followed by GsMTx4 treatment. *n* = 8. Shown are mean values ± SEM. Statistical significance was determined by unpaired Student’s *t* test or the Mann-Whitney *U* test. **P < 0.01; ***P < 0.001.

To assess the in vivo role of PIEZO1 in myofibroblasts during pulmonary fibrosis, *Postn-CreER^T2^; Piezo1^fl/fl^* (*Pn-Piezo1–KO*) mice were generated and subjected to bleomycin injury (Figure 4A). Deletion of *Piezo1* in Postn^+^ cells strongly reduced bleomycin-induced pulmonary fibrosis (Figure 4, B–D). *Pn-Piezo1–KO* mice also showed a strong reduction of hydroxyproline in the lung, which is a major component of collagen (Figure 4E). We compared the expression of fibrotic genes in association with myofibroblast activation and found *Postn, Fn1, Col1a1, Col3a1, Acta2,* and *Tgfb1* to be dramatically decreased in *Pn-Piezo1–KO* mice (Figure 4, F–H and Supplemental Figure 4, A–C). Deletion of *Piezo1* in Postn^+^ cells also strongly suppressed profibrotic TGF-β–Smad2/3 pathway activation (Figure 4H and Supplemental Figure 4, D and E). Compared with the WT littermates, the lung function, including airway resistance and lung compliance, was improved in *Pn-Piezo1–KO* mice with bleomycin injury (Figure 4I). Also, *Pn-Piezo1–KO* mice had improved survival rate and body weight compared with WT littermates (Supplemental Figure 4, F and G). Consistently, *Piezo1* deletion with *Col1a2-CreER^T2^*, a cre line mainly targeting fibroblast-traced myofibroblasts, also moderately attenuated bleomycin-induced lung fibrosis (Supplemental Figure 5, A–D). To further validate these phenotypes, we subjected WT mice to GsMTx4, a pharmacological blocker for PIEZO1. To this end, GsMTx4 was intratracheally administrated during the process of bleomycin-induced fibrosis on 1, 7, and 14 d.p.i. and, thereafter, lungs were analyzed (Supplemental Figure 6A). Similarly, systematic histological analysis revealed that inhibition of PIEZO1 with GsMTx4 significantly reduced the fibrotic area in the lung (Figure 4, J and K). Immunostaining also confirmed that there is a striking reduction in Postn^+^ cell accumulation in the GsMTx4 treated lungs (Figure 4L). Moreover, GsMTx4 treatment improved the survival rate, body weight loss, and hydroxyproline content upon injury (Supplemental Figure 6, B-D).

### PIEZO1 promotes myofibroblast activation and lung fibrosis via YAP/TAZ.

**Figure 5:**
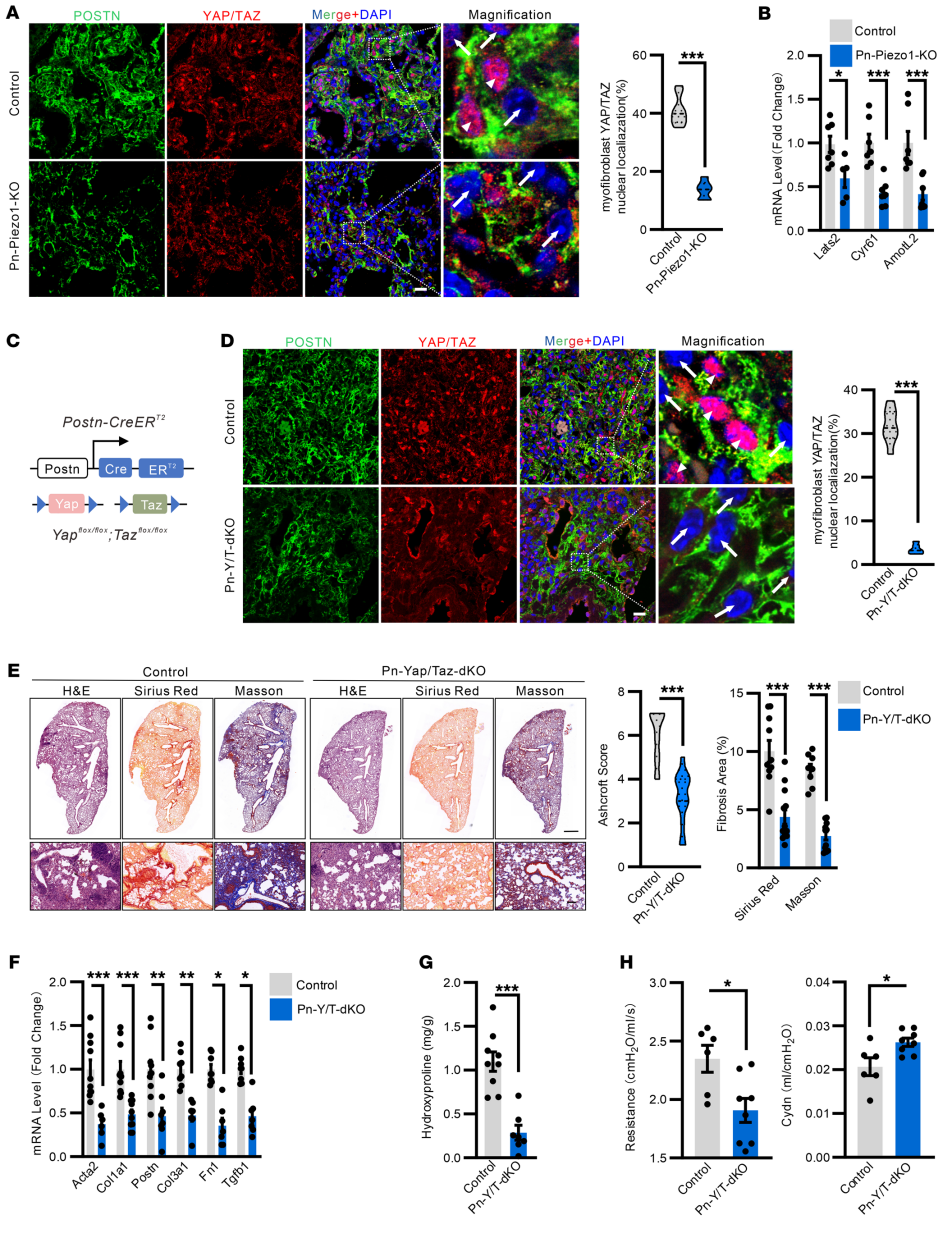
Myofibroblast *Yap/Taz* deletion ameliorate lung fibrosis. (**A**) Representative images (*left*) and quantification (*right*) of YAP/TAZ expression in the Postn^+^ cells in the lung sections of *Pn-Piezo1*-*KO* and control (*Piezo1^fl/fl^*) mice after BLM challenge. High magnification images show nuclear (arrowheads) and extranuclear (arrows) YAP/TAZ. Percentage of nuclear YAP/TAZ in GFP-positive cells was quantified. Scale bar: 20 μm. *n* = 6–8. (**B**) Real-time qPCR analysis of *Lats2*, *Cyr61*, and *AmotL2* mRNA expression levels in the lung homogenates of *Pn-Piezo1*-*KO* and control mice at 21 d.p.i. *n* = 5–7. (**C**) Schematic representation showing the genetic strategy for the generation of the *Postn-CreER^T2^;Yap^fl/fl^;Taz^fl/fl^* mice (*Pn-Yap/Taz*-*dKO*). (**D**) Representative images (*left*) and quantification (*right*) of YAP/TAZ expression in the Postn^+^ cells in the lung sections of *Pn-Yap/Taz*-dKO and control (*Yap^fl/fl^;Taz^fl/fl^*) mice after BLM challenge. High magnification images show nuclear (arrowheads) and extranuclear (arrows) YAP/TAZ. Scale bar: 20 μm. *n* = 6–8. (**E**) (*left*) Representative images of H&E, Sirius Red, and Masson staining in the lung sections of *Pn-Yap/Taz*-*dKO* and control mice after BLM challenge. (Scale bar: 1 mm, top; 100 μm, bottom). (*right*) Quantification of the Ashcroft score, Sirius red fibrosis, and Masson fibrosis area. *n* = 8–13. (**F**) Real-time qPCR analysis of *Acta2, Col1a1*, *Postn*, *Col3a1, Fn1,* and *Tgfb1* mRNA expression levels in the lung of *Pn-Yap/Taz*-*dKO* and control mice after BLM challenge. *n* = 6–10. (**G**) Hydroxyproline content in the lungs of *Pn-Yap/Taz*-*dKO* and control mice after BLM challenge. *n* = 7–9. (**H**) Lung resistance and Cdyn analysis of *Pn-Yap/Taz*-*dKO* and control mice at 21 d.p.i. *n* = 6–8. Shown are mean values ± SEM. Statistical significance was determined by unpaired Student’s *t* test or the Mann-Whitney *U* test. **P < 0.01; ***P < 0.001.

Yes-associated protein (YAP) and transcriptional coactivator with PDZ-binding motif (TAZ) are mechanosensitive transcriptional cofactors that have been implicated as drivers for organ fibrosis. Reanalysis of single-cell transcriptomics (39, 40) indicates that both *Yap* and *Taz* expression are strongly upregulated in myofibroblasts from patients with IPF (Supplemental Figure 7, A and B). Here, we hypothesized that YAP/TAZ might serve as important downstream mechanotransducer mediating PIEZO1-induced mechanoresponses in myofibroblasts. Indeed, we observed predominant YAP and TAZ nuclear localization in the activated Postn^+^ cells in the lungs of bleomycin-treated control mice (Figure 5A). However, deletion of *Piezo1* resulted in significant suppression of YAP and TAZ nuclear localization in myofibroblasts (Figure 5A). Quantitative PCR showed that expression of *Yap/Taz* target genes such as *Lats2, Cyr61,* and *AmotL2* were downregulated by *Piezo1* deletion in myofibroblasts (Figure 5B and Supplemental Figure 4, A–C). To directly study role of YAP/TAZ in myofibroblasts and to avoid potential compensation, *Postn-*CreER^T2^*; Yap^fl/fl^; Taz^fl/fl^* mice (*Pn-Yap/Taz–dKO*) were generated and exposed to bleomycin to address YAP and TAZ function in myofibroblasts during lung fibrosis (Figure 5C and Supplemental Figure 7C). We first confirmed the loss of *Yap/Taz* expression in myofibroblasts from *Pn-Yap/Taz–dKO* mice with bleomycin-induced fibrosis (Figure 5D and Supplemental Figure 7D). At 21 d.p.i., deletion of both *Yap* and *Taz* in myofibroblasts strongly attenuated lung fibrosis and suppressed the expression of key myofibroblast activation and profibrotic genes, including *Acta2*, *Col1a1, Postn, Fn1, Col3a1,* and *Tgfb1* (Figure 5, E and F). The phosphorylation of Smad2/3 was also strongly reduced in myofibroblasts from bleomycin-injured *Pn-Yap/Taz–dKO* mice (Supplemental Figure 7, E and F). Hydroxyproline levels in the homogenate from the lung was also decreased upon myofibroblasts *Yap/Taz* deletion (Figure 5G). Furthermore, *Pn-Yap/Taz-dKO* mice showed considerably improved respiration function, survival rate, and weight gain compared with the littermate controls (Figure 5, G and H and Supplemental Figure 7, G and H).

### Piezo1 or Yap/Taz loss induces altered cell state in myofibroblasts.

**Figure 6:**
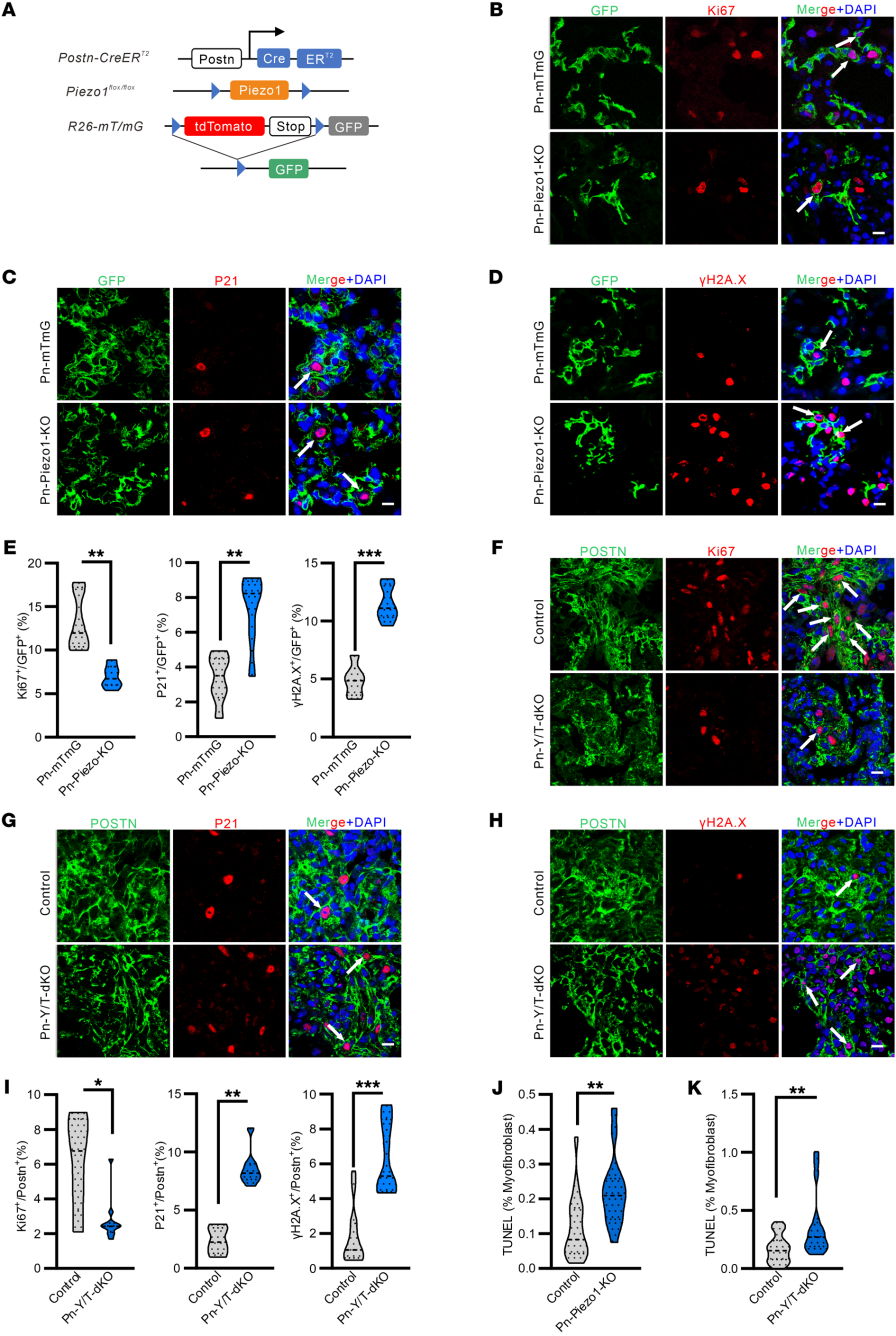
PIEZO1 and YAP/TAZ regulate myofibroblast proliferation and survival. (**A**) Schematic representation showing the genetic strategy for generation of the *Postn-CreER^T2^; Piezo1^fl/fl^; mT/mG* mice (*Pn-Piezo1*-*mT/mG*). (**B**–**D**) Representative images of Ki67 (**B**), P21 (**C**), and γ-H2A.X (**D**) expression in the GFP^+^ cells in the lung sections of *Pn-Piezo1*–*KO*, *mT/mG,* and *Pn-mT/mG* mice after BLM challenge. Scale bar: 20 μm. (**E**) Quantification of Ki67^+^, P21^+^, and γ-H2A.X^+^ in Postn^+^ myofibroblasts in the lung sections of *Pn-Piezo1*–*KO*, *mT/mG,* and *Pn-mT/mG* mice after BLM challenge. *n* = 6. The double-positive cells were defined by a GFP membrane around the Ki67, P21, or γ-H2A.X-positive nuclear. (**F**–**H**) Representative images of Ki67 (**F**), P21 (**G**), and γ-H2A.X (**H**) expression in the GFP^+^ cells in the lung sections of *Pn-Yap/Taz*-*dKO* and control mice after BLM challenge. Scale bar: 20 μm. (**I**) Quantification of Ki67^+^, P21^+^, and γ-H2A.X^+^ in Postn^+^ myofibroblast in the lung sections of *Pn-Yap/Taz*-*dKO* and control mice after BLM challenge. *n* = 5–11. (**J** and **K**) Quantification of TUNEL^+^ myofibroblast percentage in the lung sections of *Pn-Piezo1*-*KO*, *mT/mG* (**J**, *n* = 14–17) and *Pn-Yap/Taz*-*dKO* (**K**, *n* = 17–28) mice after BLM challenge. Shown are mean values ± SEM. Statistical significance was determined by unpaired Student’s *t* test or the Mann-Whitney *U* test. **P < 0.01; ***P < 0.001.

To explore how PIEZO1 alters the myofibroblast cell state in vivo, we crossed *Postn-Piezo1–KO* mice with the *mT/mG* reporter line to simultaneously trace the fate of myofibroblasts lacking *Piezo1* (Figure 6A). To this end, *Postn-CreER^T2^;Piezo1^fl/fl^;mTmG* mice (KO) or *Postn-CreER^T2^;mTmG* mice (Control) were injected with tamoxifen upon bleomycin injury and cell states including proliferation, survival, or cellular stress of GFP lineage myofibroblasts were analyzed. At 21 d.p.i., histological images of lung sections stained for Ki67 indicated that a significant proportion of GFP^+^ myofibroblasts were proliferating, while *Piezo1* deletion greatly reduced the number of proliferative GFP^+^ cells (Figure 6, B and E and Supplemental Figure 4C). Meanwhile, a minor population of GFP^+^ myofibroblasts from injured *Postn-CreER^T2^; mT/mG* (Control) mice showed positive for P21 or γ-H2A.X, which are markers for cell cycle arrest and DNA damage. However, the number of P21 or γ-H2A.X–positive GFP^+^ cells were all significantly increased after myofibroblast *Piezo1* deletion (Figure 6, C–E and Supplemental Figure 4C). Similarly, the number of proliferative myofibroblasts was significantly decreased in fibrotic lungs from *Pn-Yap/Taz-dKO* mice at 21 d.p.i (Figure 6, F and I), while the cell number of P21 or γ-H2A.X–positive myofibroblasts was increased in *Pn-Yap/Taz-dKO* mice(Figure 6, G–I). Moreover, even though activated myofibroblasts are resistant to apoptosis, both *Pn-Piezo1–KO* and *Pn-Yap/Taz–dKO* mice showed relatively more TUNEL^+^ myofibroblasts in the injured lung compared with their littermate controls (Figure 6, J and K). These data suggest that, in the context of bleomycin treatment, *Piezo1* or *Yap/Taz* deletion in myofibroblasts attenuates lung fibrosis, at least partly, via shifting the myofibroblasts from a proliferative into a stressed and apoptotic state.

### Mechanical response is impaired in PIEZO1-null myofibroblasts.

**Figure 7:**
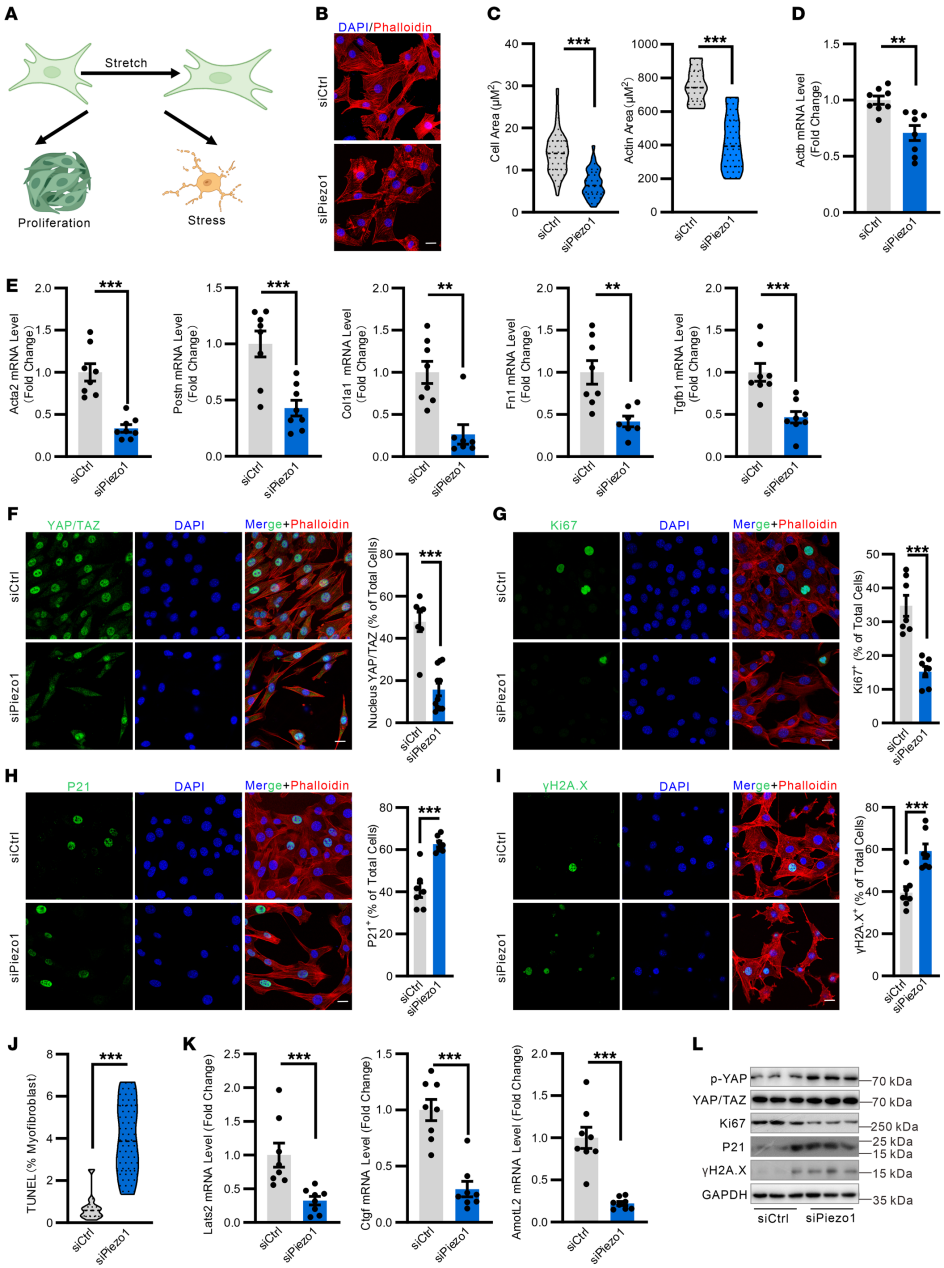
*Piezo1* loss impedes mechanical response in myofibroblasts. (**A**) Schematic diagram of the experimental design for the myofibroblast stretch. NIH3T3 cells were differentiated to myofibroblasts by stimulation with 5 ng/mL Tgf-β1 for 72 hours. Then, myofibroblasts were subjected to 10% stretch for 12 hours at 1 Hz. (**B** and **C**) Representative images of cell morphological change and quantification of cell area and actin area change of myofibroblasts under cyclic stretch. Scale bar: 20 μm. *n* = 12–13. (**D**) Real-time qPCR analysis of actin mRNA expression levels in myofibroblasts under cyclic stretch. *n* = 8. (**E**) Real-time qPCR analysis of *Acta2, Postn, Col1a1, Fn1*, and *Tgfb1* mRNA expression levels in myofibroblasts under cyclic stretch. *n* = 7–8. (**F**–**I**) Representative images and quantification of YAP/TAZ (**F**), Ki67 (**G**), P21 (**H**), and γ-H2A.X (**I**) expression in myofibroblasts under cyclic stretch. Cells were costained with phalloidin. Scale bar: 20 μm. *n* = 7–11. (**J**) Quantification of TUNEL^+^ percentage in myofibroblasts under cyclic stretch. *n* = 10–11. (**K**) Real-time qPCR analysis of *Lats2, Ctgf,* and *AmotL2* mRNA expression levels in myofibroblasts under cyclic stretch. *n* = 8. (**L**) Western blot analysis of the indicated protein levels under cyclic stretch. GAPDH was used as the internal control. Shown are mean values ± SEM. Statistical significance was determined by unpaired Student’s *t* test or the Mann-Whitney *U* test. **P < 0.01; ***P < 0.001.

To directly explore the role of PIEZO1 in the mechanical responses of myofibroblasts in vitro, mouse NIH3T3 and human MRC5 fibroblasts were differentiated into myofibroblasts by TGF-β and cultured on silicon membranes designed for uniaxial cell stretching (Figure 7A). As expected, mechanical force rapidly influenced cell shape, and uniaxial stretch triggered a robust cytoskeletal remodeling and cell spreading in control cells (Figure 7, B and C). However, those mechanical responses were severely impaired in *Piezo1*-deficient myofibroblasts, most of which had a shrinking and round appearance (Figure 7, B and C). In parallel, knockdown of *Piezo1* strongly inhibited stretch-induced upregulation of the cytoskeleton gene *Actb* as well as of profibrotic genes including *Postn, Fn1, Col1a1,* *Acta2*,and *Tgfβ1*, (Figure 7, D and E). Furthermore, stretch induced a predominant YAP/TAZ nuclear localization in the control cells, while *Piezo1*-deficient myofibroblasts displayed limited YAP/TAZ nuclear localization, and expression of *Yap/Taz* target genes, including *Lats2, Cyr61,* and *AmotL2,* were suppressed upon *Piezo1* knockdown (Figure 7, F and K). Transfection of TGF-β differentiated MRC5 myofibroblast cells with mutant *YAP^5SA^*, the LATS1/2 phosphorylation sites of YAP were mutated from S to A, resulting in a full reversal of the *PIEZO1* knockdown–induced suppression of YAP target genes and cellular phenotypes (Supplemental Figure 8, A–G), suggesting YAP as a key downstream factor for PIEZO1-mediated mechanotransduction in myofibroblasts. Moreover, both stretch- and Yoda1-induced YAP nuclear localization in myofibroblasts are markedly prevented by Rhosin (RhoA inhibitor) and Y27632 (ROCK inhibitor), suggesting a role of RhoA/ROCK pathway in linking PIEZO1-YAP/TAZ mechanotransduction (Supplemental Figure 9, A–D). A prior study has shown that, in epithelial cells, PIEZO1 mediates stretch-induced proliferation (41). In line with this, stretch-induced proliferation is greatly reduced in myofibroblasts lacking *PIEZO1*. (Figure 7G, Supplemental Figure 8D, and Supplemental Figure 9E). In contrast, the numbers of myofibroblasts costained with P21, γ-H2A.X, and TUNEL were strongly increased after *PIEZO1* knockdown (Figure 7, H–J and Supplemental Figure 8, E and F). These perturbed mechanoresponses were further validated by Western blot analysis. (Figure 7L and Supplemental Figure 9, F–I). Consistent with an impaired mechanoresponse, *Piezo1* knockdown blocked stretch-induced phosphorylation of Smad2/3 in activated myofibroblasts (Supplemental Figure 9J).

## Discussion

Myofibroblasts are the key cell type that drives tissue fibrosis in various organs. In lung injury, myofibroblasts are synergistically activated by biochemical and mechanical stimuli to drive fibrosis by excessive remodeling and accumulation of the ECM, which leads to perturbed mechanical homeostasis in the lung (7, 23, 25). However, very little is known about the mechanism by which myofibroblasts sense the mechanical cues in the lung. In this study, by using the *Postn-CreER^T2^* mice, we report that Postn is a reliable and distinctive marker for myofibroblasts and that ablation of Postn^+^ cells after injury strongly improved lung fibrosis and respiration function. We discovered that the mechanosensitive cation channel PIEZO1 is highly expressed in Postn^+^ cells and plays a vital role in the mechanoactivation of Postn^+^ cells and lung fibrosis. Conditional *Piezo1* deletion or pharmacological PIEZO1 inhibition in myofibroblasts dramatically improved lung fibrosis by suppressing myofibroblasts activation and proliferation. Mechanistically, loss of *PIEZO1* led to disruption of actin organization, prevention of YAP/TAZ nuclear localization, and cell stress. Furthermore, myofibroblast-specific *Yap/Taz* deletion fully recapitulated the protective phenotypes of myofibroblast-*Piezo1–KO* mice in the bleomycin injury model (Supplemental Figure 10).

Depending on organ type and the nature of insult, pathological myofibroblasts can originate from multiple cell types (18). In lung injury, the origin of myofibroblasts is still controversial and the debated progenitors include resident fibroblasts, bone-marrow-derived circulating fibrocytes, pericytes, AEC2 cells, ADRP^+^ lipofibroblasts, Axin2^+^ progenitors, and Gli1^+^ PDGFR-β^+^ mesenchymal cells (3, 6, 11, 13, 14, 16–18, 42). Prior lineage tracing studies (12) in the lung have ruled out the direct contribution of epithelial cells in myofibroblast precursors and suggest that a substantial portion of myofibroblasts derived from multiple stromal cells. Much of the confusion is due to the substantial overlap of marker genes between myofibroblasts with other cells, especially fibroblasts, smooth muscle cells, and pericytes (42). Fate mapping studies (16, 17) suggested that Gli1^+^ cells give rise to approximately 37% of myofibroblasts, while PDGFR-β^+^ cells give rise to approximately 70% of myofibroblasts at 14 days after bleomycin injury. Notably, both Gli1 and PDGFR-β identify different mesenchymal cells, which are committed to multiple lineages in organ fibrosis, respectively (16, 17). A recent scRNA-seq analysis identified that a collagen-producing Cthrc1^+^ (collagen triple helix repeat containing 1) fibroblast emerges in fibroblastic foci from injured lungs (43). Tsukui et al. (10) identified Scube2^+^ alveolar fibroblasts as the dominant source of multiple emergent fibroblast subsets after fibrotic lung injury. They further demonstrate that CTHRC1^+^ fibroblasts are significant contributors to fibrosis. However, conflicting data exist for both a pro- and antifibrotic role of CTHRC1 (44, 45). Our studies indicate that Postn marks myofibroblasts in the injured lung, regardless of their prior origin, and they can be traced by *Postn-CreER^T2^*. In line with this, our recent studies (19, 22) have demonstrated that Postn labels almost all myofibroblasts in the fibrotic liver and heart which was independently confirmed by Kanisicak et al (21). Meanwhile, we observed an abundant expression of Postn in the fibrotic foci of lungs from patients with IPF. Furthermore, diphtheria toxin–mediated depletion of Postn^+^ pulmonary myofibroblasts resulted in the suppression of profibrotic gene expression. Most intriguingly, ablation of Postn^+^ myofibroblasts resulted in a remarkable fibrosis resolution in the injured lung. Since no adverse effects on lung morphology and function were detected, strategies allowing for the selective inhibition of the Postn^+^ cell population seem a promising target for future therapies. Nevertheless, the reanalysis of single-cell data from fibrotic lungs of both mice and humans revealed the expression of Postn in other lung cells, including fibroblasts. Consequently, there is an acknowledgment of the limitation for further, systematic validation in order to confirm Postn as a biomarker for lung myofibroblasts.

Tissue fibrosis is accompanied by disrupted mechanical homeostasis resulting in pathological ECM deposition and stiffening (36, 46). Under continued mechanical stress, myofibroblasts are fully activated to acquire a high contractile and proliferative state by neoexpressing α-SMA and ECM proteins, which promote positive feedback loops that propagate profibrotic remodeling (25, 36). As a mechanosensitive cation channel activated by various physical forces, PIEZO1 has been implicated in fibrosis in various organs including skin, heart, kidney, and liver (47–53). Prior studies (54) suggested that activation of PIEZO1 induces ECM synthesis, fibroblast differentiation, macrophage infiltration, and EMT in fibrotic tissues. However, little is known about the expression and function of PIEZO1 in myofibroblasts and its contribution to pulmonary fibrosis. We generated the *Piezo1-CreER;GFP* knock-in mice to genetically monitor endogenous PIEZO1 expression in the bleomycin-injured lung. In addition to previously reported vascular endothelial and smooth muscle cells, we found PIEZO1 to be abundantly expressed in Postn^+^ myofibroblasts in the fibrotic foci of lung starting from day 3 after bleomycin injury. In line with this, recent single cell analyses of fibrotic murine lung (39, 40) also confirmed a strong expression of PIEZO1 in Postn^+^ myofibroblasts. Nevertheless, we acknowledge the need for careful protein-level validation to confirm PIEZO1 expression in myofibroblasts. Linked to these findings, we generated the *Postn-CreER^T2^; Piezo1^fl/fl^; mT/mG* mice to simultaneously target PIEZO1 in myofibroblasts and trace its fate during the course of lung fibrosis.

PIEZO1 can be activated by various mechanical forces acting on cellular membranes, including fluid shear stress, osmotic swelling, compression, and stretch of cell membranes (27, 55–57). PIEZO1 has been implicated in fibroblast-to-myofibroblast transition and the secretion of extracellular matrix (58). However, how PIEZO1 is mechanically activated in myofibroblasts is not known. In fact, our patch-clamp recording of activated myofibroblasts revealed the existence of large stretch-induced currents, with remarkable slow deactivation kinetics. Knockdown of *Piezo1* by siRNA almost abolished the stretch-induced current, suggesting a key role of PIEZO1 in mechanosensing in myofibroblasts. While the mechanism of slow inactivation kinetics is unclear, it is possible that PIEZO1 might operate in response to chronic mechanical cues in lung myofibroblasts. Mechanical stimuli set up a positive feedback loop through the ECM that is capable of sustaining tissue stiffness and progressive fibrosis (23, 46). Recent data (50, 59, 60) have implicated PIEZO1 as a mechanosensor for stiffness in diverse cells including macrophages, DRG neurons, and osteoblasts. In addition to the matrix component, lung cells, including myofibroblasts, are mechanically stretched with the alveolar expansion during each breath cycle. In fact, we show that, in cultured myofibroblasts, PIEZO1-mediated stretch-induced cellular responses, including actin remodeling and cell spreading. A previous study (41) showed that stretch triggered rapid proliferation of epithelial cells by activation of the ERK1/2–cyclin B pathway in a PIEZO1-dependent manner. Similarly, our data demonstrate that activation of PIEZO1 by mechanical stretch induced proliferation in cultured myofibroblasts. We also found a substantial proportion of Ki67-positive proliferating myofibroblasts in the bleomycin-injured lung. Interestingly, the number of Ki67^+^ cells was dramatically reduced in *Pn-Piezo1–KO* mice. Meanwhile, we observed that myofibroblasts lacking *PIEZO1* are more vulnerable to stretch-induced cell cycle arrest and DNA damage. In light of this and previous studies, we might predict PIEZO1 to act as an essential stretch sensor to maintain the proliferative state and to control the cell numbers of myofibroblasts. Nevertheless, it remains to be uncovered whether other forces generated at the cell-cell or cell-ECM interface is involved in activation of PIEZO1 in myofibroblasts. In addition, other ion channels including PIEZO2, TRPV4, and TRPC6 may also be involved in mechanosensation (61–63). A recent report suggested PIEZO2 as a critical mechanoreceptor for myofibroblast differentiation in pulmonary fibrosis (64). Our data suggest that inhibition of PIEZO1 with GsMTx4 significantly reduced the fibrotic area in the lung; however, GsMTx4 also targets other cation mechanosensitive channels, including TRPV4 (65–67), and, thus, the specific targets and in vivo antifibrotic mechanism of GsMTx4 merit further investigation.

The mechanosensitive Hippo-YAP/TAZ pathway is widely implicated in the pathogenesis of organ fibrosis (68, 69), and our data suggest that PIEZO1-induced YAP/TAZ signaling drives myofibroblast activation in pulmonary fibrosis. During fibrotic remodeling, YAP/TAZ is activated by increased tissue stiffness caused by ECM deposition, which, in turn, sets up a profibrotic feedback loop that amplifies YAP/TAZ signaling (23, 46, 68). In addition to stiffness, YAP/TAZ signaling is involved in stretch-induced mechanotransduction (70, 71). We found that mice with myofibroblast-specific *Yap/Taz* deletion recapitulated the phenotype of induced myofibroblast-specific *Piezo1* deficiency and resulted in a profound reduction of myofibroblast accumulation and lung fibrosis, which was accompanied by attenuated ECM deposition and improved lung function. A recent study (72) suggests that *Yap/Taz* deletion with *Col1a2-CreER* attenuates bleomycin-induced lung injury and CCl_4_-induced liver fibrosis. Surprisingly, we previously provided evidence (22) that, in Postn^+^ myofibroblasts, YAP an TAZ are dispensable for both CCl_4_- and bile duct ligation–induced liver fibrosis. Importantly, prior fate mapping studies with Col1a2-CreER (73) indicated that this cre line labels injury-induced myofibroblasts but also residential fibroblasts in the healthy lung, while our lineage tracing studies demonstrate that Postn^+^ cells are not present in the health state. In the current study, we observed that bleomycin induced a predominant YAP/TAZ nuclear localization in Postn^+^ myofibroblast, an effect that was prevented by deletion of *Piezo1* in Postn^+^ myofibroblasts. Similarly, our in vitro studies suggest that stretch induces myofibroblast activation and YAP/TAZ nuclear localization, and again those effects depend on PIEZO1. In addition, PIEZO1 mediates stretch-induced upregulation of *Yap/Taz* target genes, such as *Cyr61, AmotL2*, and *Lats2* in myofibroblasts. We hypothesize that PIEZO1 might regulate YAP/TAZ partially via the RhoA/ROCK pathway, since PIEZO1-induced YAP nuclear localization is markedly inhibited by the RhoA/ROCK blockers Rhosin and Y27632. Thus, we speculate that PIEZO1 operates as an upstream regulator of YAP/TAZ in myofibroblast activation.

In summary, we demonstrate that the Postn^+^ myofibroblasts drive lung fibrosis and PIEZO1-mediated mechanosensation essential for myofibroblast activation and the development of lung fibrosis. Our findings highlight mechanical signaling as a core driver of fibrosis, and we believe that targeting Postn-expressing cells and PIEZO1 is a novel potential approach to develop effective drugs for treatment of fibrotic diseases such as IPF.

## Methods

### Sex as a biological variable.

Our study examined male and female animals; similar findings are reported for both sexes.

### Generation of transgenic mice.

All mice were backcrossed onto the C57BL/6J background for at least 8 generations. Experiments were performed with Cre-negative littermates as controls. Male and female animals at an age of 8–12 weeks were used if not stated otherwise. Mice were housed under a 12 hour light-dark cycle with free access to food and water and under specific pathogen-free conditions. *Postn-CreERT2, Piezo1-CreER, Piezo1^fl/fl^, Yap^fl/fl^, Taz^fl/fl^* mouse lines were described previously (19, 37, 74, 75). The cre reporter lines, *Gt(ROSA)26Sor^tm2Sho/^J(GFP)*, *(ROSA)26Sor^tm4(ACTB–tdTomato,–EGFP)Luo^/J (mT/mG)*, *Gt (ROSA)26Sor^tm1(HBEGF)Awai^/J* *(short i-diphtheria toxin receptor [iDTR])*, *Tg(Col1a2-cre/ERT,-ALPP)7Cpd/J* were from The Jackson Laboratory. To induce Cre recombination, 75 mg/kg of tamoxifen (MCE, HY-13757A) was injected intraperitoneally for 5 consecutive days. To ablate Postn-expressing cells, animals were treated with 75 mg/kg of tamoxifen for 5 consecutive days followed by intraperitoneal injection of 100 ng diphtheria toxin (List Labs, Cat 150) for 7 days.

### Bleomycin induction of pulmonary fibrosis in mice.

Transgenic mice (8 weeks old) were intratracheally administrated with 1 U/kg of bleomycin (BLM, Cat 7763-87-5, Yuanye Bio-Technology) dissolved in saline. Mice were euthanized at the 7th, 14th, 21st, and 60th day after BLM administration, and lungs were harvested for further study. For GsMTx4 treatment, C57 WT mice were intratracheally administrated with 1 U/kg of BLM. Then, 0.5 mg/kg of GsMTx4 (Cat HY-P1410A, MCE) was intratracheally administrated on 1, 7, and 14 d.p.i., and lungs were harvested for subsequent study. The lung tissues were harvested for Western blot, quantitative real time PCR, histology analysis, and immunofluorescence staining. All of the experimental mice were fed the same chow diet and water.

### Lung function analysis.

At 21 days after bleomycin administration, endotracheal intubation was performed after anesthetizing the mice. The mice were placed in a resistance/compliance (R/C) collection station (FinePointe RC; Buxco Research Systems) and were mechanically ventilated at a rate of 150 breaths/minute. The R/C system directly detected the lung ventilation and intrapulmonary pressure in mice, then values such as airway resistance (RI), and lung compliance (Cdyn) were derived. Lung function was recorded and calculated by FinePoint software (Buxco Electronics Ltd).

### Flow cytometry analysis.

For GFP^+^ cell isolation, mice were sacrificed at day 21 after BLM intratracheal inhalation. Lungs were harvested and cut into small fragments. The tissue pieces were collected and dissociated by 2 mg/mL collagenase II (Worthington) in HBSS on a shaker at 180 rpm for 50 minutes at 37 °C. A 12-mL syringe was used to resuspend cells in to a single-cell suspension, which was filtered through a 70-μm cell strainer (Falcon) and centrifuged at 600*g* for 5 minutes at 4°C. Erythrocytes were lysed with red cell lysis buffer (Biosharp) for 5 minutes on ice. After washing with HBSS, the cells were collected by centrifugation and resuspended in HBSS to a final density of 1 × 10^6^ cells per 100 μL. Samples were blocked with Fc blocker (BD Bioscience) for 5 minutes and stained with the corresponding antibodies. All samples were analyzed on a BD FACSCelesta flow cytometer (BD Bioscience). Data analyses were conducted using FlowJo_V10.

### Histological analysis.

Harvested lungs were inflated and placed in fresh 4% neutral-buffered formalin for 24 hours to fix at 4°C, and then washed in 1× phosphate buffered saline (PBS). For paraffin sections, lungs were dehydrated, washed, and embedded in paraffin. Following this, 5 μm sections were cut, mounted on glass, and stained with H&E (Cat ST047, Solarbio), Hete& (Cat E8090, Solarbio), Trichrome Masson’s (Catalog abs9348, Absin), and Sirius Red (Catalog DC0041, Leagene biotech). Bright-field images were taken with an Olympus microscope. The Ashcroft score ranges from 0 to 8, with higher scores indicating more severe fibrosis. Scores of 0–1 represented no fibrosis, and scores of 6–8 indicated severe fibrosis. Each field was scored for the fibrosis degree in a blinded fashion by 2 individuals. Digitized images were analyzed by Image-Pro Plus 6.0 software (Media Cybernetics).

### Cell culture and treatment.

Mouse embryonic fibroblast cell lines (NIH3T3, Cat CL-0171) and human fetal lung fibroblast (MRC-5 cell line, Cat CL-0161) were obtained from Procell, Inc. and cultured at 37°C in an atmosphere containing 5% CO_2_ and in DMEM (Gibco) supplemented with 10% FBS and 1% penicillin and streptomycin.

Cells were serum starved in FBS-free DMEM overnight and differentiation to myofibroblasts was induced by treatment with 5 ng/mL TGF-β1 (Cat HY-P70648, MCE) for 72 hours. TGF-β–induced myofibroblasts were then treated for specified time points and placed in the Cell Tank system (Cell & Force) to stretch for 12 hours at 1 Hz. The range of stretching is set to 10% of the cell size.

In some experiments, the RhoA inhibitor Rhosin (MCE, Cat HY-12646) and the ROCK inhibitor Y27632 (MCE, Cat HY-10071) were used. Briefly, MRC5 at a confluence of 80% were pretreated with 1 μM of Rhosin or Y27632 for 15 minutes followed by 1 μM of Yoda1 exposure for 30 minutes, or static or cyclic stretch for 12 hours. The cells were then harvested for analysis.

### Lentivirus infection of cells.

For the package of lentivirus of mutant *YAP^5SA^*, HEK293T cells were infected with the *YAP^5SA^* plasmid (addgene Cat 33093) and packaging vectors using PolyJet (WZ Biosciences). Following a 48-hour period, the medium was collected and filtered using a 0.45-micron filter to yield the virus-containing supernate, which was then stored at 4°C for use in cell infection. Meanwhile, MRC5 cells were cultured in fresh media and subsequently infected with lentivirus overnight. The culture media was changed 2 days after infection, and 2 g/mL puromycin (Solarbio) was added to the medium for screening successfully infected cells.

### Collagen gel contraction assay.

The collagen gel contraction assay was conducted utilizing a cell contraction assay kit (CBA-021) procured from Cell Biolabs Inc., following the manufacturer’s protocol. In summary, MRC5 cells were transfected with either scrambled siRNA or *PIEZO1* siRNA for 48 hours and subsequently resuspended in DMEM supplemented with 10% FBS at a confluence of 70%–80%. A collagen lattice was prepared by combining the cell suspension with an ice-cold collagen gel solution at a volume ratio of 1:4. Subsequently, 0.5 mL of the mixture was dispensed into each well of a 24-well plate and incubated at 37°C for 1 hour. Thereafter, 1 mL of DMEM containing 10% FBS was added atop each collagen gel lattice. After 24 hours, the plate was scanned and the gel area was analyzed using ImageJ software.

### siRNA-mediated knockdown.

Cells were transfected with siRNAs using Opti-MEM (Thermo Fisher Scientific) and Lipofectamine RNAiMAX (Invitrogen) as described previously (28). For transfection of cells*,* 20 pmoles of siRNA were mixed gently with RNAiMAX and incubated for 30 minutes at room temperature. The mixture was then added to cell culture medium. The medium was changed to fresh complete medium after 6 hours. Experiments were performed 48 hours later. siRNAs against *Piezo1* were from Genepharma. The targeted sequences of mouse siRNAs directed against RNAs encoding PIEZO1 was as follows: *Piezo1*, 5′-UCGGCGCUUGCUAGAACUUCA-3′, 5′-UGAAGUUCUAGCAAGCGCCGA-3′.

### Quantitative RT-PCR analysis.

Total RNA was isolated from myofibroblasts or lung tissues with Trizol reagent (Cat 15596018, Invitrogen). A total of 1 μg of RNA was used for cDNA synthesis with the cDNA synthesis kit (Cat 170-8891, BioRad) according to the manufacturer’s instructions. qRT-PCR was performed with universal SYBR green mix (Cat 172-5122, BioRad) on the StepOne Plus System (Applied Biosystems). 2^–ΔΔCT^ method was used to quantify the relative expression of target genes. Relative expression levels were obtained by normalization with *GAPDH* or *18S* values. Quantitative PCR primer sequences were as follows: *β-actin, 5*′*-actgtcgagtcgcgtcca-3′/5′*-atccatggcgaactggtgg-3′; *iDTR, 5*′*-ggagcacgggaaaagaaag-3*′*/5*′*- gagcccggagctccttcaca-3*′*; Lats2 5*′*-gtgcttctccgcaaagggta-3*′*/5*′*- gcccaaccagcatctcaaaga-3*′*; Amotl2, 5*′*-actgtacctaagccgaaccg-3*′*/5*′*- cttgcacacacctgcctaga-3*′*; Ctgf, 5*′*- aagctgacctggaggaaaaca-3*′*/5*′*- tgcagccagaaagctcaaac-3*′; *Col3a1, 5*′*- tgctggaaagaatggggagac-3*′*/5*′*- ggtccagaatctcccttgtcac-3*′; *Col1a1, 5*′*- ttcagggaatgcctggtgaa-3*′*/5*′*- acctttgggaccagcatca-3*′; *Postn, 5*′*- ccattggaggcaaacaactcc-3*′*/5*′*- ttgcttcctctcaccatgca-3*′; *Acta2, 5*′*- gaggcaccactgaaccctaa-3*′*/5*′*- tacatggcggggacattgaa-3*′; *Fn1, 5*′*- cgtcattgccctgaagaaca-3*′*/5*′*- aagggtaaccagttggggaa-3*′; *Tgfb1, 5*′*- gctgcgcttgcagagattaa-3*′*/5*′*- gtaacgccaggaattgttgcta-3*′*; Piezo1, 5*′*-ccttctgttgctggtgtttg-3*′*/5*′*-gtcctggtccaacctctgg-3*′*; 18S, 5’-gtaacccgttgaaccccatt-3*′*/5*′*-ccatccaatcggtagtagcg-3*′.

### Immunofluorescence staining.

For cryosections, tissue was embedded in optimal cutting temperature compound (‘OCT’, Tissue-Tek, Cat 4583), cryosectioned at –20°C (5 μm sections), and transferred onto adhesive slides. For paraffin-embedded tissue sections, lung sections (5 μm) were dewaxed and dehydrated. Antigen retrieval was done by boiling the slides in 10 mmol/L sodium citrate (pH 6.0) at 98°C for 10 minutes. For cell samples, cells were fixed with 4% PFA for 15 minutes. Tissue or cell slides were blocked with 5% BSA (Cat V900933, Sigma-Aldrich) and permeabilized with 0.3% Triton X-100 (Cat T8200, Solarbio) for 1 hour at room temperature. Samples were stained with antibodies against Rage (1:100, Cat ab216329, Abcam), CD31 (1:100, Cat MA513188, Thermo Fisher Scientific), α-SMA-cy3 (1:100, Cat C6198, Sigma-Aldrich), Pdgfr-α (1:100, Cat AF1062, R&D), Pdgfr-β (1:100, Cat 14-1402-82, Invitrogen), Desmin (1:100, Cat 5332S, CST), Postn (1:100, Cat AF2955, R&D Systems), PIEZO1 (1:100, Cat 15939-1-AP, ProteinTech), Ki67 (1:100, Cat ab16667, Abcam), P21 (1:100, Cat 10355-1-AP, ProteinTech), γH2AX (1:400, Cat ab81299, Abcam), YAP/TAZ (1:100, Cat 8418S, CST), Phalloidin-594 (1:100, Cat 8953S, CST), Collagen1 (1:100, Cat AF7001, Affinity), overnight at 4°C. The next day, slides were incubated with corresponding AlexaFluor-conjugated isotype-specific secondary antibodies [Alexa Fluor 488 goat anti-rabbit IgG(H+L), Cat A11008; Alexa Fluor 594 goat anti-rabbit IgG(H+L), Cat A11012; Alexa Fluor 594 donkey anti-goat IgG(H+L), Cat A11058; Alexa Fluor 647 donkey anti-mouse IgG(H+L), Cat A31571; Alexa Fluor 647 donkey anti-rabbit IgG(H+L), Cat A31573; Alexa Fluo 647 Goat anti-Rat IgG (H+L) Cat A21247; and Alexa Fluo 488 donkey anti-Goat IgG (H+L), Cat A11055, all from Invitrogen] (1:200, Invitrogen) or TUNEL assay solution (Cat C1086, Beyotime) at room temperature for 1 hour Nuclei were counterstained with DAPI (1 μg/mL, Thermo Fisher Scientific) for 5 minutes at room temperature. After washing 3 times with PBS, the samples were mounted with mounting media (Vector Laboratories). Immunofluorescence imaging was performed using a Leica TCS SP5 confocal microscope.

### Western blot.

Myofibroblasts and isolated lungs were lysed by RIPA buffer (Cell Signaling, Cat 9806) supplemented with protease and phosphatase inhibitors (Cat 04906837001, Roche). Lysates were centrifuged at 11,752*g* at 4°C for 10 minutes. Supernatants were then subjected to SDS-PAGE and transferred to PVDF membranes. After blocking in 5% skim milk for 1 hour, blots were probed using the below primary antibodies: Fibronectin1 (1:1000, Cat F3648, Sigma-Aldrich), Postn (1:1000, Cat AF2955, R&D Systems), α-SMA (1:1000, Cat AF1032, Affinity), GFP (1:1000, Cat 66002-1-IG, ProteinTech), YAP/TAZ (1:1000, Cat 8418S, CST), Phospho-YAP (1:1000, Cat 13008S, CST), Ki67 (1:1000, Cat ab16667, Abcam), P21 (1:1000, Cat 10355-1-AP, ProteinTech), and γH2AX (1:1000, Cat ab81299, Abcam) was used as the loading control. Blots were developed using the ECL detection system (BioRad).

### Determination of [Ca^2+^]_i_.

For the determination of the intracellular Ca^2+^ concentration, myofibroblasts were loaded with 5 μM Ca^2+^-sensitive dye Fluo-4 AM (AAT Bioquest, Cat 20551) in HBSS supplemented with 20 mM HEPES for 30 minutes at 37°C and were then washed with HBSS 3 times at room temperature. Live cell images were acquired with an IX81 microscope (Olympus) at a frequency of 1 Hz. Fluo-4 fluorescence was measured by using excitation at 488 nm and emission collected at 500–550 nm. Fluorescent intensity was measured with FlexStation 3 (Molecular Devices).

### Determination of cell membrane tension.

Membrane tension was determined using the membrane tension probe Flipper-TR, as previously described (30, 74, 76). Briefly, myofibroblasts were stained with 1 μM of the membrane tension probe Flipper-TR (Tebu-bio, Cat SC020) for 30 minutes at 37°C. Cells were then washed 3 times with HBSS and imaged with a Leica-SP8 FLIM microscope. Excitation was stimulated using a pulsed 488 nm laser (Laser kit WLL2+pulse picker, Leica Microsystems) operating at 80 MHz, and the emission signal was collected from 549 to 655 nm with acousto-optical beam splitter (AOBS) using a gated hybrid (HyD SMD) detector and a TimeHarp 300 TCSPC Module and Picosecond Event Timer (PicoQuant). SymPhoTime 64 software (PicoQuant) was used to fit fluorescence decay data. To extract lifetime information, the photon histograms from membrane regions were fitted with a double exponential, and 2 fluorescence emission decay times (τ1 and τ2) were extracted. The longest lifetime with the higher fit amplitude τ1 was used to report membrane tension.

### Electrophysiology.

Whole cell-patch clamp recordings were performed at room temperature using an EPC10/2 amplifier with Pulse software (HEKA Electronik GmbH) as previously described (30, 74). Pipette resistance was between 3 and 4 MΩ, and membrane potential was clamped at –70 mV. Normal external solution contained 140 mM NaCl, 5 mM KCl, 1.8 mM CaCl_2_,1mM MgCl_2_, 10 mM Hepes, and 10 mM glucose, pH 7.4. The intracellular pipette solution contained 95 mM L-aspartate, 40 mM CsCl, 1 mM CaCl_2_, 1 mM MgCl_2_, 10 mM Hepes, and 0.1 mM GTP, pH 7.2. Local low flow at indicated shear rate was generated with a multichannel microperfusion system (LEAD-2, LONGER). Myofibroblasts were exposed to 3 μM GdCl_3_ (Cat 10138-52-0, Sigma-Aldrich) and to mechanical stretch stimulation using a fire-polished glass pipette (Poking). Whole cell current was analyzed with IGOR Pro software (WaveMetrics).

### Single-cell RNA-seq dataset processing.

The raw data sets of public single-cell RNA-seq (scRNA-seq) previously published by Adams et al. (39) and Peyser et al. (42) were obtained from the Gene Expression Omnibus (GEO) database with the accession numbers GSE136831 and GSE129605, respectively. The data were processed using the Seurat R package, version 4.3.0.1. For GSE129605; cells with fewer than 400 detected genes or more than 20% mitochondrial genes were excluded from the analysis (RunUMAP function of Seurat, dims.use = 1:20). For GSE136831, the analysis was performed by constructing a Seurat object using the expression matrix data and metadata provided by the authors with the CreateSeuratObject function for downstream analysis (RunUMAP function of Seurat, dims.use=1:15). Violin plots were generated using ggplot2 version 3.5.1, and UMAP plots overlaid with gene expression levels were generated using Seurat.

### Statistics.

All statistical analyses were performed with GraphPad Prism 9. Trial experiments or experiments done previously were used to determine sample size with adequate statistical power. Samples were excluded in cases where RNA/cDNA quality or tissue quality after processing was poor (below commonly accepted standards). Data are presented as mean ± SEM. Comparisons between 2 groups were performed with unpaired 2-tailed Student’s *t* test and the Mann-Whitney U test. Comparisons between multiple groups were determined by 2-way ANOVA followed by Bonferroni multiple comparison tests. *P* ≤ 0.05 was considered to be statistically significant.

### Study approval.

Studies using human samples were approved by the ethics committee of Xi’an Jiaotong University (No.XJTU1AF2023LSK-152) and conform to the guidelines of the 2000 Helsinki declaration. Written informed consent was obtained from all participants before their participation.

### Data availability.

Values for all data points in graphs are reported in the Supporting Data Values file. The data associated with this paper are available upon request to the corresponding authors. The single-cell RNA-seq data of lung for human and mouse are from the NCBI Gene Expression Omnibus under the accession number GSE136831 and GSE129605.

## Author contributions

LX, TL, YC, and YH designed the study, performed experiments, analyzed data, and wrote part of the manuscript. ZS, SL, BW, AS, HT, YL, GL, CW, JXJY, YX, FC, JL, and BZ helped with both in vivo and in vitro experiments. JS, NW, SO, YY, and ZY supervised part of the study and discussed data. SW designed and supervised the study, discussed data, and wrote the manuscript; and all authors commented on the manuscript.

## Supplementary Material

Supplemental data

Unedited blot and gel images

Supporting data values

## Version 1 06/02/2025

Electronic publication
