# Supplementary material for: PIEZO1 mediates periostin+ myofibroblast activation and pulmonary fibrosis in mice
Source: J Clin Invest. 2025 Jun 2;135(11):e184158. doi: 10.1172/JCI184158 (PMC12126248; doi:10.1172/JCI184158)
Supplement: Supplemental data [file jci-135-184158-s222.pdf]

Supplemental Material

Manuscript

PIEZO1 mediates Postn⁺ myofibroblast activation and pulmonary fibrosis

(Xu et al.)

Supplemental Figures

Supplemental Figure 1

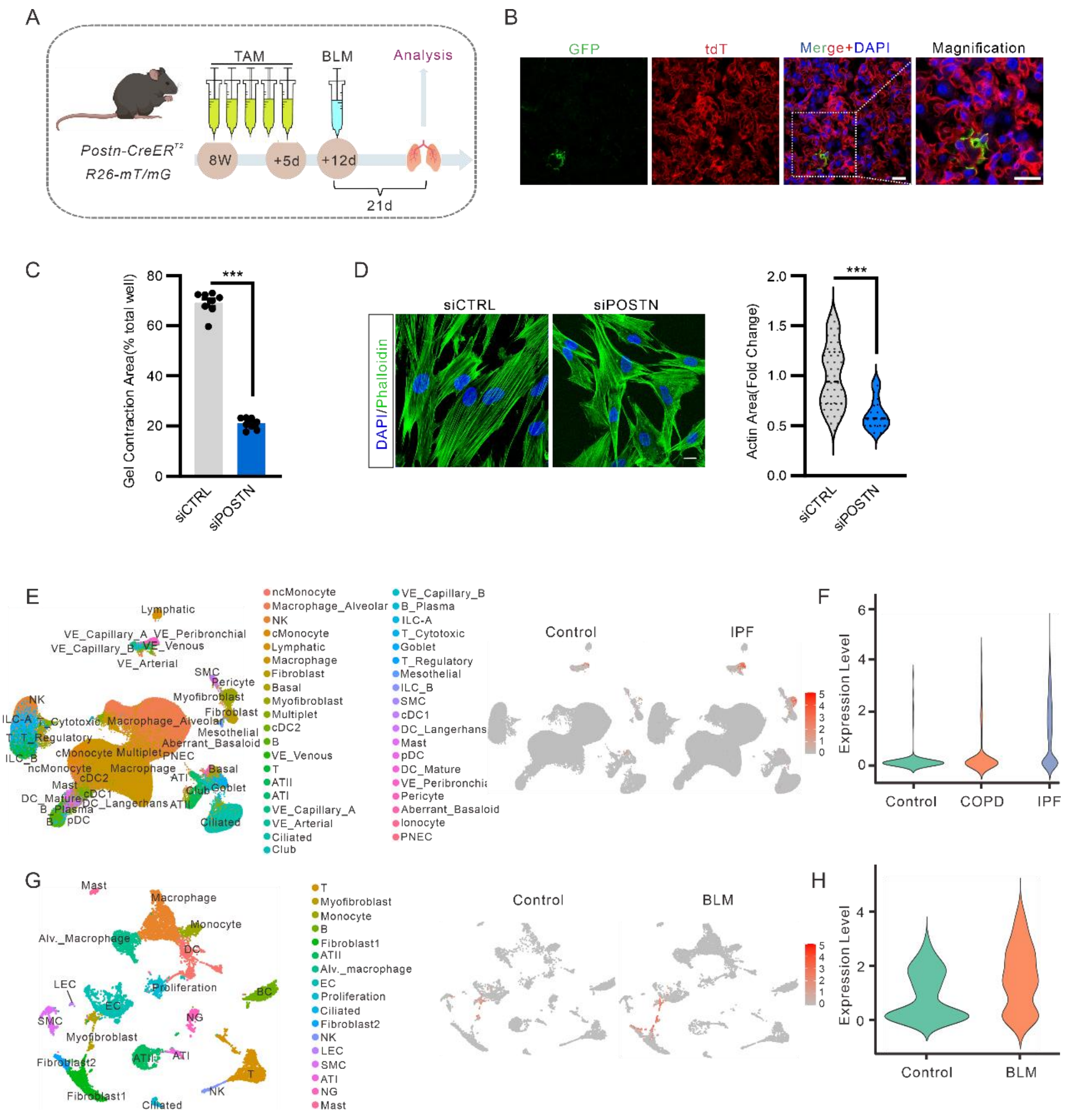

Supplemental Figure 1. Periostin expression and function in lung.

A. Schematic diagram of the experimental design. *Postn-CreER*^{T2}*; mT/mG* mice were injected with tamoxifen for 5 consecutive days, after 7 days wash out, mice were subjected to a single intratracheal inhalation of BLM. **B**. Representative images of Postn^{+} GFP cells in *Postn-CreER*^{T2}*; mT/mG* mice after tamoxifen treatment and BLM challenge. Immunofluorescent staining showing DAPI, tdTomato and GFP single channels in addition to a merged image. Scale bar, 20 μm. n=6. **C**. MRC5 were differentiated into myofibroblasts via 5 ng/mL of TGFβ, and then transfected with scrambled siRNA or POSTN siRNA for 48h. Cells were then harvested for evaluation of the size of collagen gel contraction. n=8. **D**. Representative images of stress fiber and quantification of actin area in myofibroblasts with POSTN knockdown. Scale bar, 20 μm. n=13. **E-F** Single cell transcriptomics analysis of published human dataset GSE136831. (**E**) Uniform Manifold Approximation and Projection (UMAP) representation of cell types from IPF donor lungs, and POSTN expression in control and IPF lungs. In the subject plot, each color depicts a distinct subject. (**F**) Violin plot showed POSTN expression levels in myofibroblast from COPD, IPF and control lungs. **G-H**. Single cell transcriptomics analysis of published mouse dataset GSE129605. UMAPs showed cell types of BLM-induced fibrotic mouse lungs, and POSTN expression(**G**). Violin plot showed POSTN expression levels in myofibroblast from control and fibrotic lungs (**H**). Shown are mean values ± SEM. Statistical significance was determined by unpaired Student's t test or the Mann-Whitney U test. ***P < 0.001.

Supplemental Figure 2

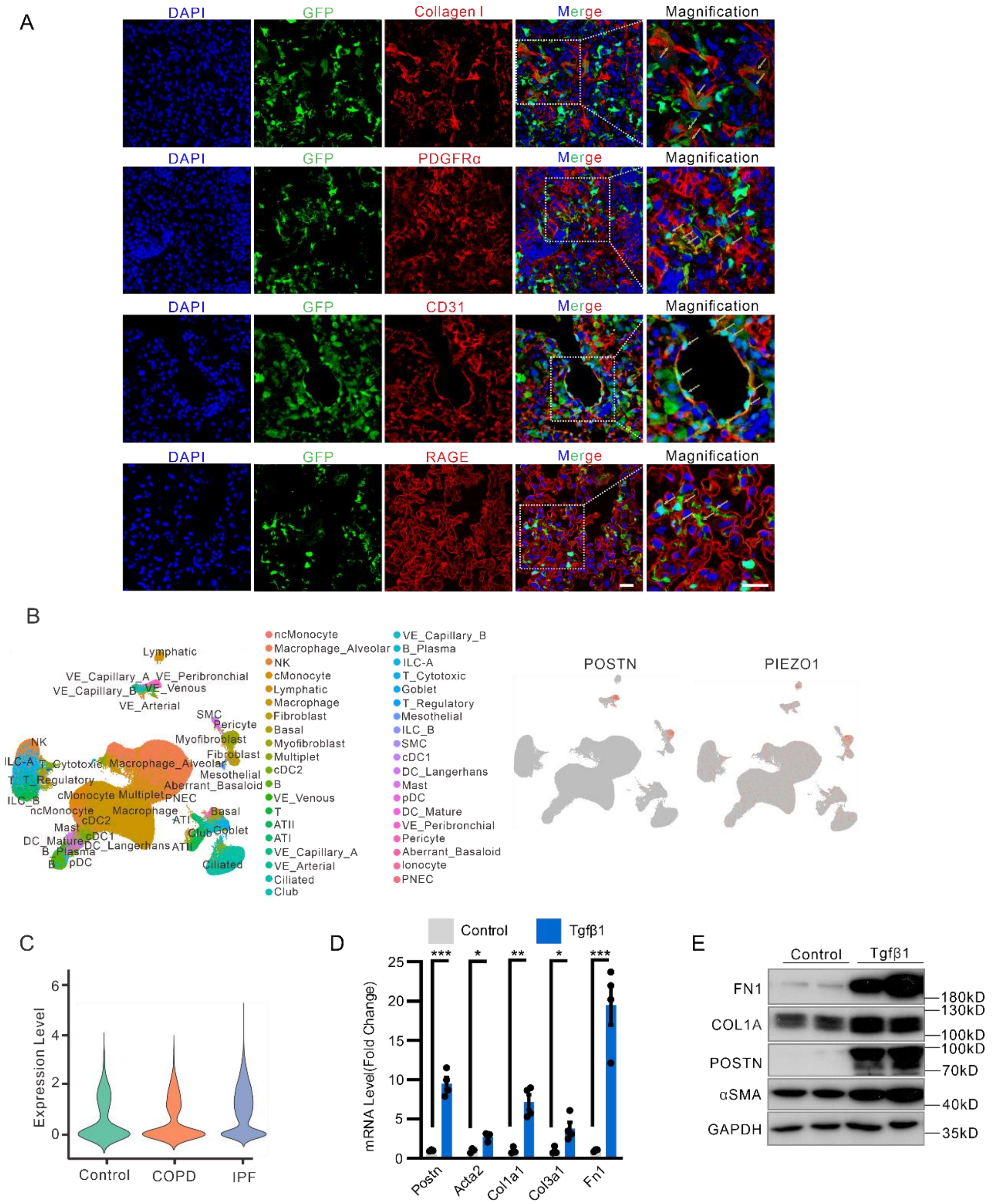

Supplemental Figure 2. PIEZO1-expressing cell lineage tracing in lung fibrosis and *in vitro* myofibroblast differentiation.

A. Representative images of GFP expression in the Collagen I^+, PDGFRα^+, $CD31^+$, or $RAGE^+$ cells in the lung sections of *Piezo1-CreER*;*R26-GFP* mice at 21 d.p.i.. Scale bar, 20 μm. **B**. Single cell transcriptomics analysis of POSTN and PIEZO1 expression in published human dataset GSE136831. **C**. Violin plot showed PIEZO expression levels in myofibroblast from COPD, IPF and control patient lungs. **D**. Real-time qPCR analysis of *Postn, Acta2, Col1a1, Col3a1* and *Fn1* mRNA expression levels in NIH3T3 cell with TGFβ stimulation for 72 h. n=3-4. **E**. Western blot analysis of the indicated protein levels in NIH3T3 cell with TGFβ stimulation for 72 h. Statistical significance was determined by unpaired Student's t test or the Mann-Whitney U test. *$P < 0.05$ was considered significant; **$P < 0.01$; ***$P < 0.001$.

Supplemental Figure 3

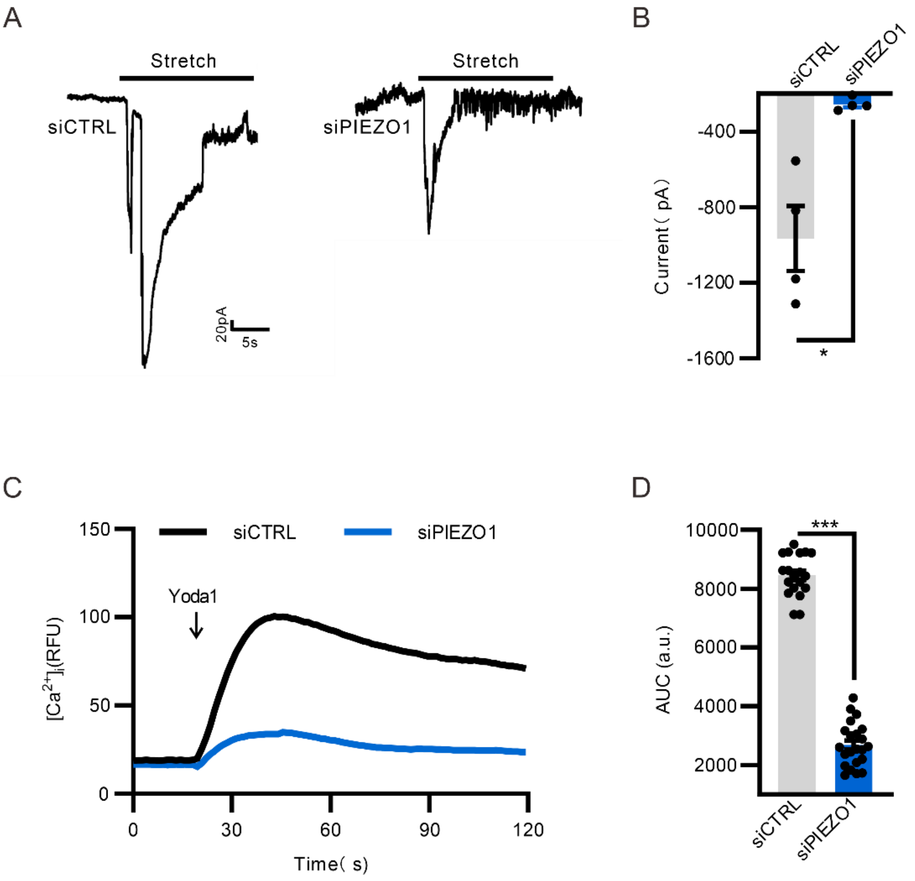

Supplemental Figure 3. Mechanosensitive current and Yoda1-induced Ca^{2+} response in myofibroblast.

A. MRC-5 cells were differentiated to myofibroblasts by stimulation with 5 ng/mL TGFβ for 72 h. Representative cell-attached patch clamp traces of stretch-activated currents in myofibroblasts transfected with control siRNAs or with siRNAs against *PIEZO1*. The holding potential was -70 mV and the membrane was stretched by pulses of negative pressure with a 10 mm Hg increment. **B**. Statistical analysis of 4 independent recordings. **C**. Fluo-4-loaded myofibroblasts transfected with control siRNAs or with siRNAs against *PIEZO1* were exposed to 1 μM Yoda1, and (**D**) $[Ca^{2+}]_i$ was determined as fluorescence intensity (RFU, relative fluorescence units); line indicates the addition of Yoda1. Bar diagrams show the area under the curve (AUC) of the Ca^{2+} transient. n=19-22. Shown are mean values ± SEM. Statistical significance was determined by unpaired Student's t test or the Mann-Whitney U test. *P < 0.05 was considered significant; ***P < 0.001.

Supplemental Figure 4

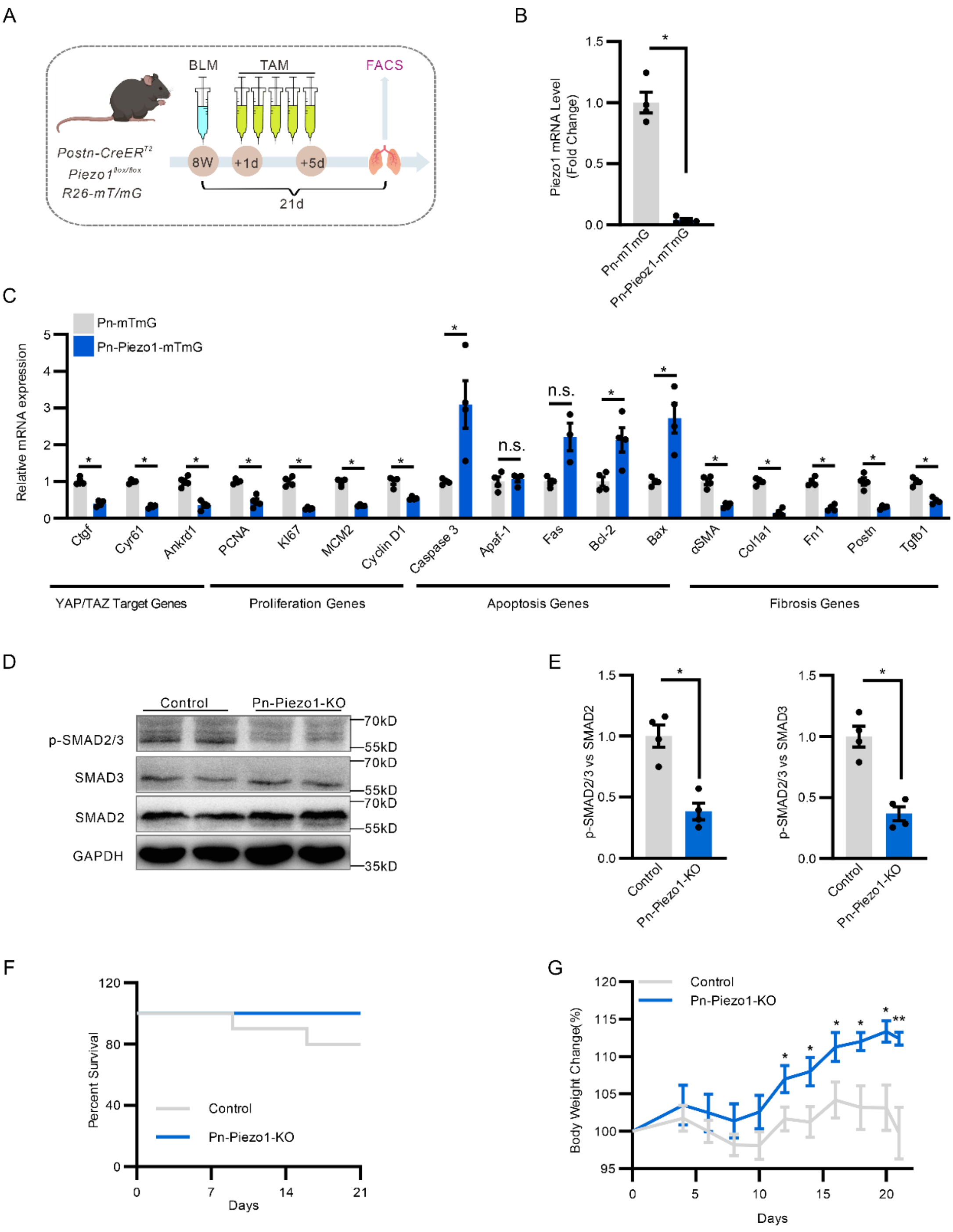

Supplemental Figure 4. PIEZO1 promotes myofibroblast Smad2/3 activation and lung injury.

A. Schematic diagram of the experimental design. *Postn-CreER*^{T2}*; Piezo1*$^{flox/flox}$ mice (*Pn-Piezo1-KO*) were challenged with a single intratracheal inhalation of BLM followed by injection with tamoxifen for 5 consecutive days. After 15 days, the mice were euthanized for subsequent analysis. **B**. Real-time qPCR analysis of *Piezo1* mRNA levels in myofibroblasts isolated from *Pn-Piezo1-mT/mG* and *Pn-mT/mG* mice. n=4. **C**. Real-time qPCR analysis of apoptosis/proliferation/fibrosis related genes and *YAP/TAZ* target gene mRNA levels in myofibroblasts isolated from *Pn-mT/mG* or *Pn-Piezo1-mT/mG* mice. n=3-4. **D-E**. Western blot analysis and quantification of the indicated protein levels in lung homogenates of *Pn-Piezo1-KO* and control mice at 21 days d.p.i. n=4. **F-G**. Survival percentage (**F**) and body weight change percentage (**G**) of *Pn-Piezo1-KO* and control mice at 21 days d.p.i. n=7-10. Shown are mean values ± SEM. Statistical significance was determined by unpaired Student's t test or the Mann-Whitney U test. n.s, no significance;**P* < 0.05 was considered significant; ***P* < 0.01.

Supplemental Figure 5

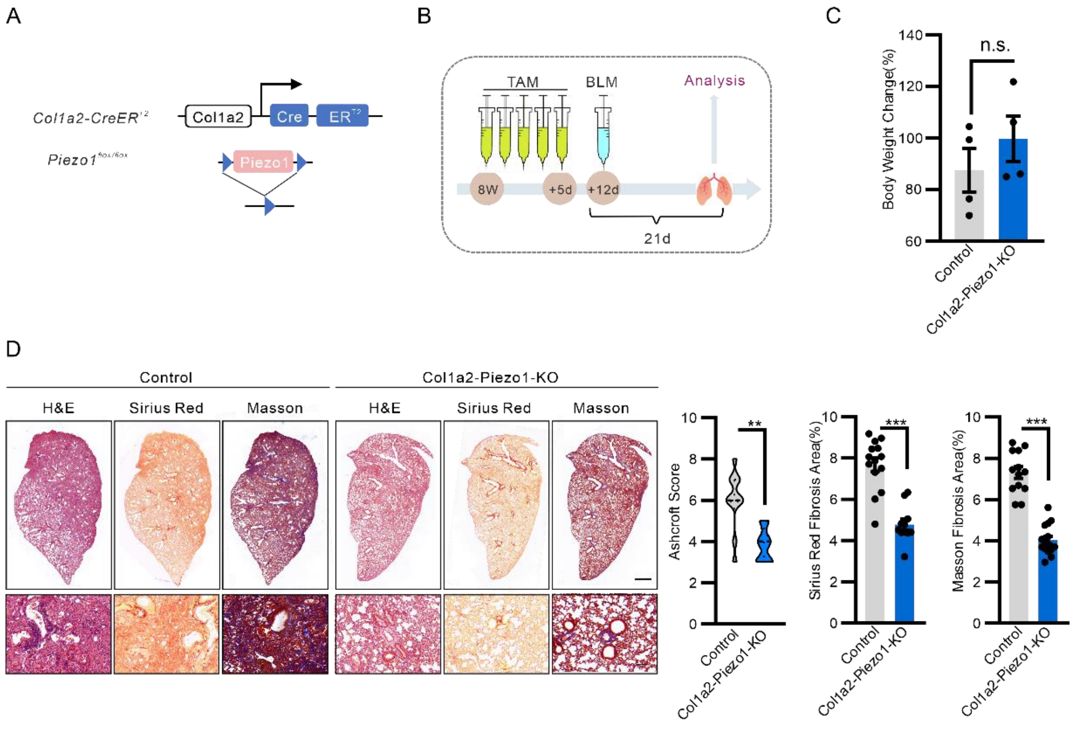

Supplemental Figure 5. Loss of *Piezo1* in Col1a2⁺ myofibroblast improves lung fibrosis.

A. Schematic representation showing the genetic strategy for generation of the *Col1a2-CreER^{T2}; Piezo1$^{flox/flox}$* mice (*Col1a2-Piezo1-KO*). **B**. Schematic diagram of the experimental design. *Col1a2-Piezo1-KO* mice were injected with tamoxifen for 5 consecutive days followed by a single intratracheal inhalation of BLM challenge. **C**. Body weight change percentage of *Col1a2-Piezo1-KO* and control (*Piezo1$^{flox/flox}$*) mice at 21 days d.p.i. n=4. **D**. (*left*) Representative images of H&E, Sirius Red, and Masson staining in the lung sections of *Col1a2-Piezo1-KO* and control mice. Scale bar, 1 mm (up),100 μm(down). (*right*) Quantification of the Ashcroft score, Sirius red fibrosis and Masson fibrosis area. n=9-15. Shown are mean values ± SEM. Statistical significance was determined by unpaired Student's t test or the Mann-Whitney U test. ***P* < 0.01; ****P* < 0.001.

Supplemental Figure 6

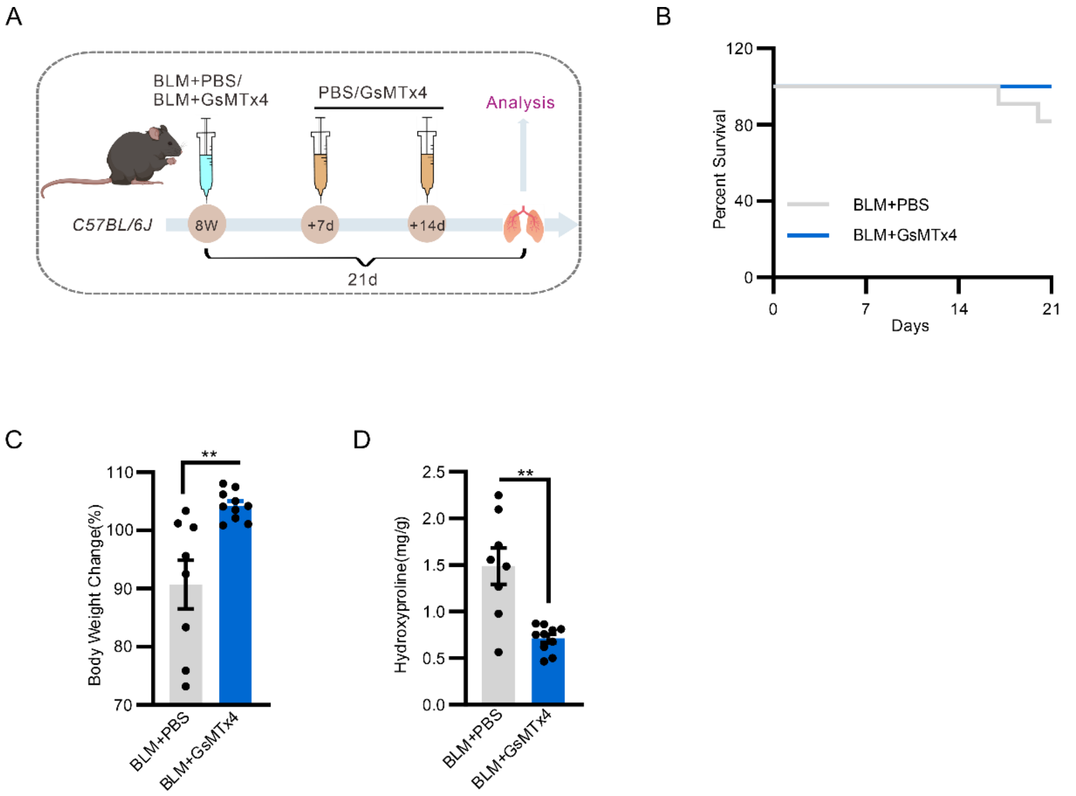

Supplemental Figure 6. Pharmacological inhibition of PIEZO1 improves survival and body weight loss in bleomycin injury.

A. Schematic diagram of the experimental design. 8 weeks old of C57BL/6J mice were challenged with BLM followed by PBS or GsMTx4 treatment. **B-C**. Percentage of survival (**B**) and body weight change (**C**) of mice subjected to BLM exposure followed by GsMTx4 or PBS treatment. n=8-10. **D**. Hydroxyproline content in the lungs of mice treated with BLM followed by PBS or GsMTx4 treatment. n=8-10. Shown are mean values ± SEM. Statistical significance was determined by unpaired Student’s t test. ***P* < 0.01.

Supplemental Figure 7

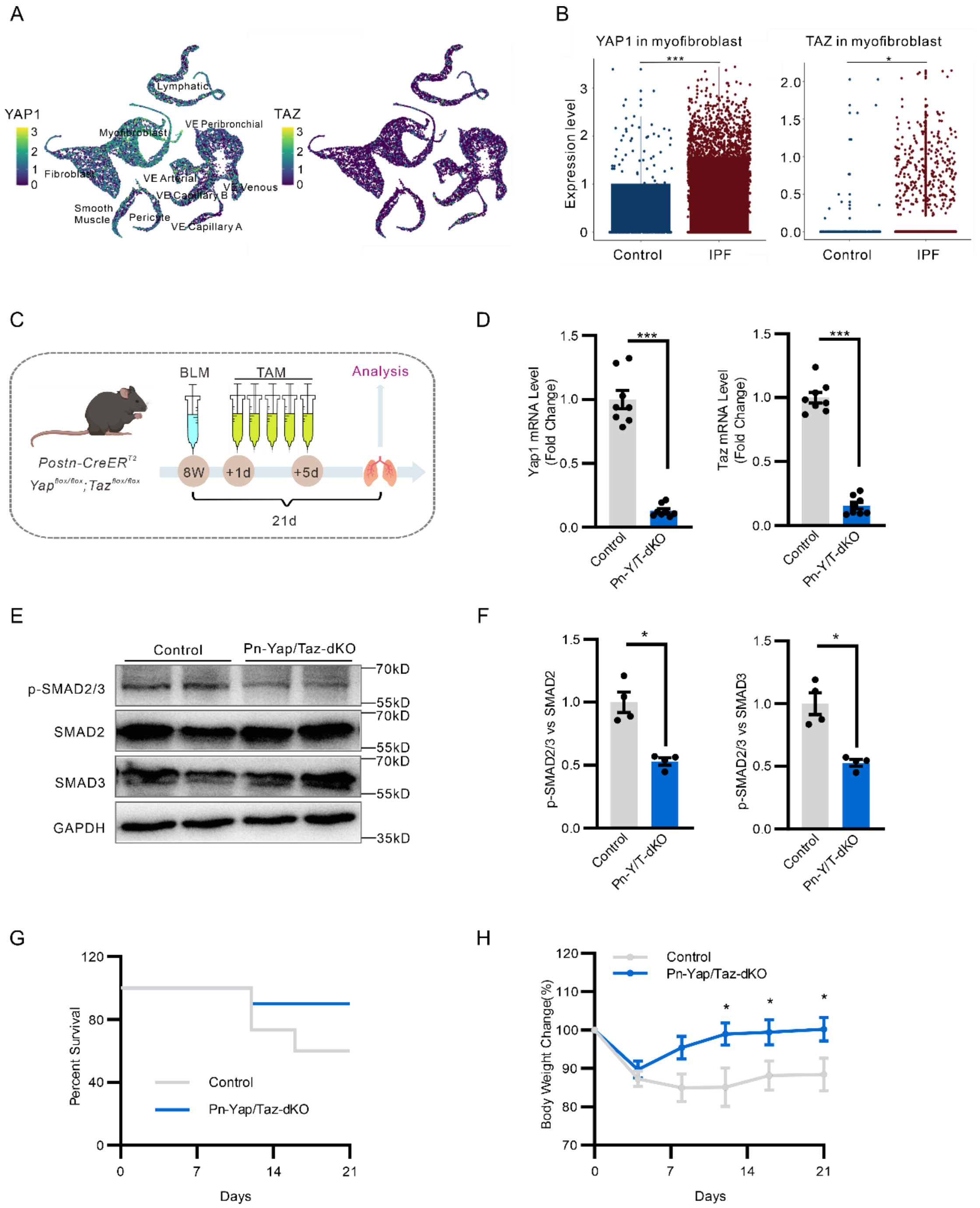

Supplemental Figure 7. Myofibroblast *Yap/Taz* deletion inhibits Smad2/3 activation and improves survival.

A-B. Re-analysis of single cell transcriptomics in human lung. Single cell transcriptomics in lung from IPF and control patients (http://www.ipfcellatlas.com). (**A**). UMAPs showed *YAP1* and *TAZ* expression in stromal cells. (**B**). Scatter bar graph showed *YAP1*and *TAZ* expression levels in myofibroblast from IPF and control patients. **C**. Schematic diagram of the experimental design. *Postn-CreER^{T2};Yap$^{flox/flox}$;Taz$^{flox/flox}$* mice (*Pn-Yap/Taz-dKO*) were challenged with BLM followed by injection with tamoxifen for 5 consecutive days. After 15 days, the mice were euthanized for subsequent analysis. **D**. Real-time qPCR analysis of *Yap1 and Taz* mRNA levels in myofibroblasts isolated from control (*Yap$^{flox/flox}$;Taz$^{flox/flox}$*) and *Pn-Yap/Taz*-dKO mice. n=8. **E-F**. Western blot analysis (**E**) and quantification (**F**) of the indicated protein levels in lung homogenates of *Pn-Yap/Taz-dKO* and control mice at 21 d.p.i. n=4. **G-H**. Percentage of survival (**G**) and body weight change (**H**) of *Pn-Yap/Taz-dKO* and control mice at 21 d.p.i. n=7-9. Shown are mean values ± SEM. Statistical significance were determined by unpaired Student's t test or the Mann-Whitney U test. *P < 0.05 was considered significant; ***P < 0.001.

Supplemental Figure 8

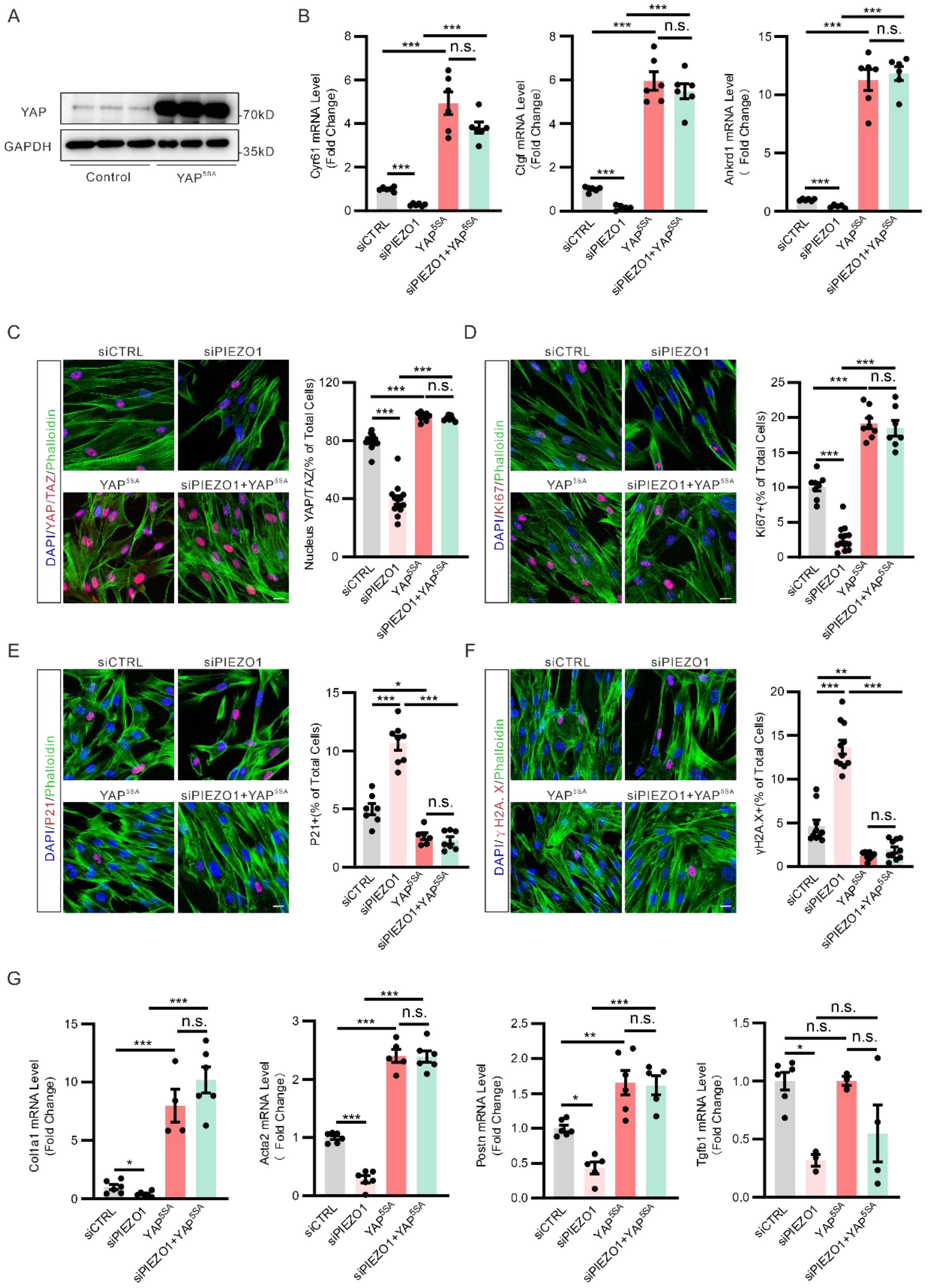

Supplemental Figure 8. Mutant *YAP^{5SA}* reversals phenotypes induced by *PIEZO1* knockdown.

A. Western blot analysis of the indicated protein levels in MRC-5 with mutant *YAP^{5SA}* expression. n=3. **B**. Real-time qPCR analysis of Cyr61, Ctgf and Ankrd1 mRNA expression levels in TGFβ-induced MRC-5, which were transfected with siCTRL or siPIEZO1 for 48h, followed by transfected with Lenti-Control or Lenti-*YAP^{5SA}* for 48 h. n=5-6. **C-F**. Representative images and quantification of YAP/TAZ (**C**), Ki67 (**D**), P21 (**E**), and γH2A.X (**F**) expression in TGFβ-induced MRC-5 under 10% of cyclic stretch. Cells were costained with phalloidin. Scale bar, 20 μm. n=6-13 **G**. Real-time qPCR analysis of *Col1a1*, *Acta2*, *Postn*, and *Tgfb1* mRNA expression levels in TGFβ-induced MRC-5, which were transfected with siCTRL or siPIEZO1 for 48h, followed by transfected with Lenti-Control or Lenti-*YAP^{5SA}* for 48 h. n=3-6. Shown are mean values ± SEM. Statistical significance was determined by 2-way ANOVA followed by Bonferroni multiple comparison tests. n.s, no significance; *P < 0.05; **P < 0.01; ***P < 0.001.

Supplemental Figure 9

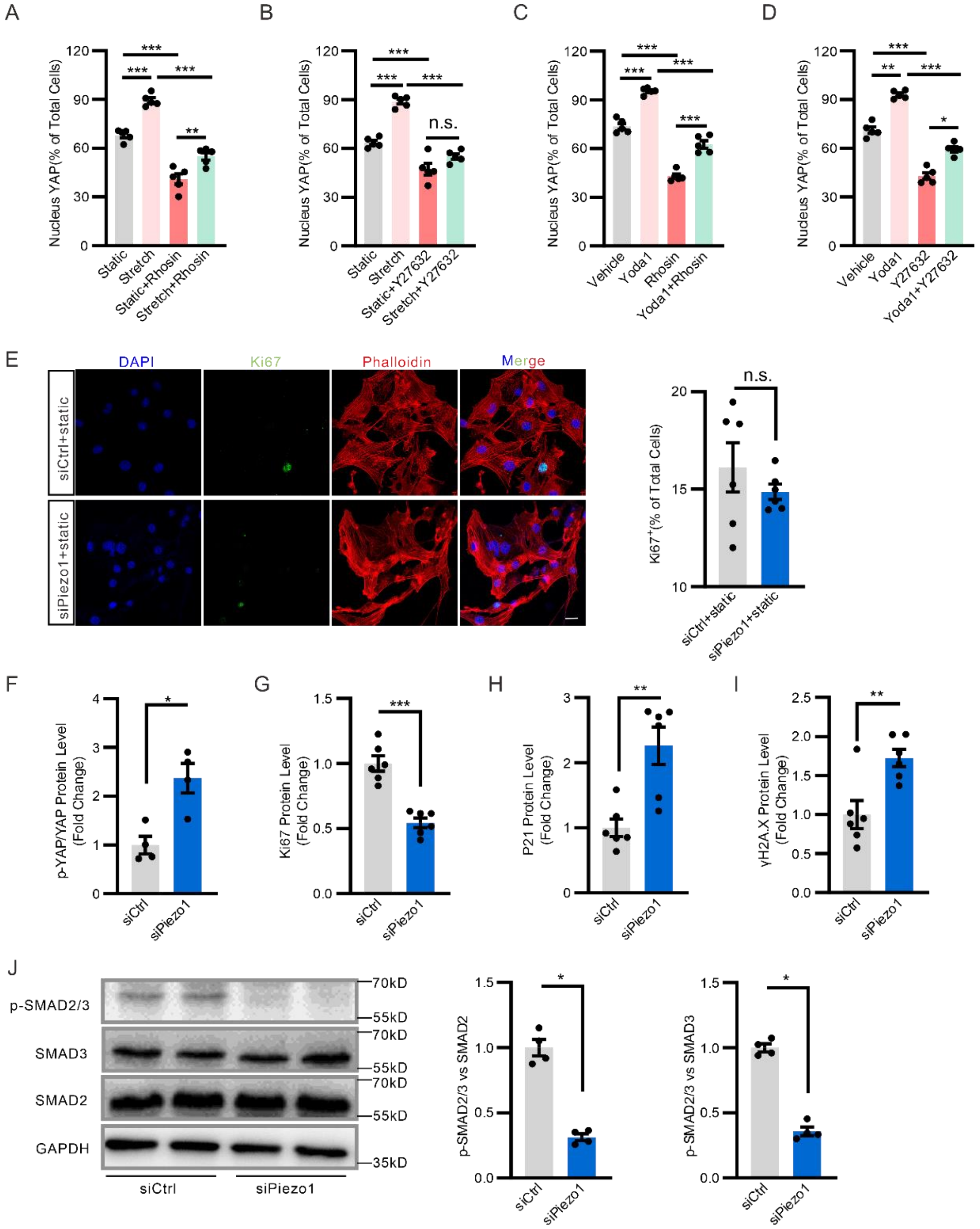

Supplemental Figure 9. PIEZO1 mediates stretch-induced mechano-signaling.

A-B. Quantification of YAP nuclear localization in myofibroblasts pretreated with Rhosin (RhoA inhibitor,1 μM) or Y27632 (ROCK inhibitor,1 μM) followed by static or cyclic stretch for 12h. n=5. **C-D**. Quantification of YAP nuclear localization in myofibroblasts pretreated with Rhosin (1 μM) or Y27632 (1 μM) followed by 1 μM of Yoda1 exposure for 30min. n=5. **E**. Representative images and quantification of Ki67 expression in myofibroblasts under static condition. Cells were co-stained with phalloidin. Scale bar, 20 μm. n=6. **F-I**. Quantification of the indicated protein levels in myofibroblasts with *Piezo1* knockdown followed by cyclic stretch treatment. n=4-6. **J**. Western blot analysis (*left*) and quantification (*right*) of the indicated protein levels in myofibroblasts with *Piezo1* knockdown followed by cyclic stretch treatment. n=4. Shown are mean values ± SEM. Statistical significance was determined by unpaired Student's t test or the Mann-Whitney U test, or 2-way ANOVA followed by Bonferroni multiple comparison tests. *P < 0.05 was considered significant; **P < 0.01; ***P < 0.001.

Supplemental Figure 10

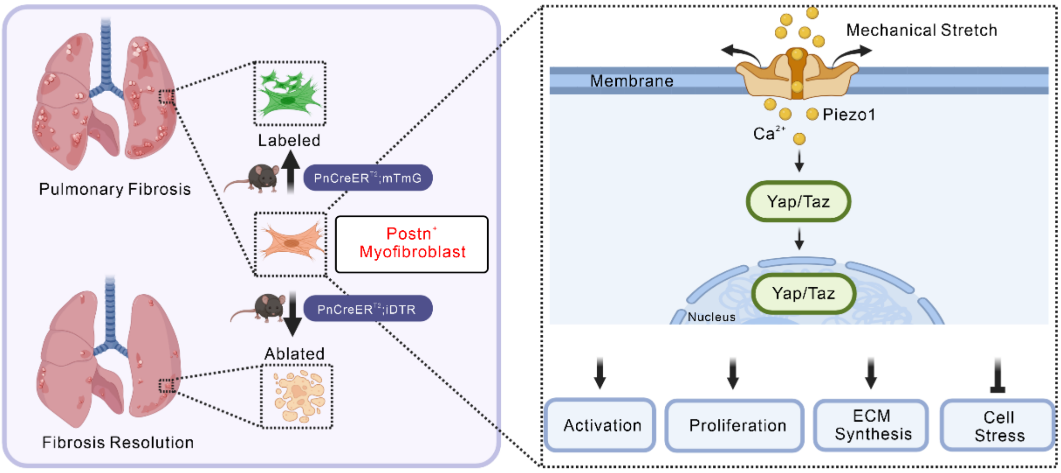

Supplemental Figure 10. PIEZO1 plays a vital role in mechano-activation of Postn^{+} myofibroblast and lung fibrosis.

Schematic representation showing that Postn^{+} cells identify myofibroblasts in bleomycin-induced lung fibrosis, and that ablation of Postn^{+} myofibroblasts after injury strongly improved lung fibrosis. The activation of myofibroblasts in the lung is dependent on PIEZO1-mediated mechanosensation. Mechanistically, the deletion of *Piezo1* inhibits the nuclear localization of YAP/TAZ, myofibroblast activation, proliferation, and extracellular matrix synthesis, and promotes cellular stress.

.
