# Supplementary material for: PIEZO1 mediates periostin+ myofibroblast activation and pulmonary fibrosis in mice
Source: J Clin Invest. 2025 Jun 2;135(11):e184158. doi: 10.1172/JCI184158 (PMC12126248; doi:10.1172/JCI184158)

Full unedited blot/gel for Figure 1D

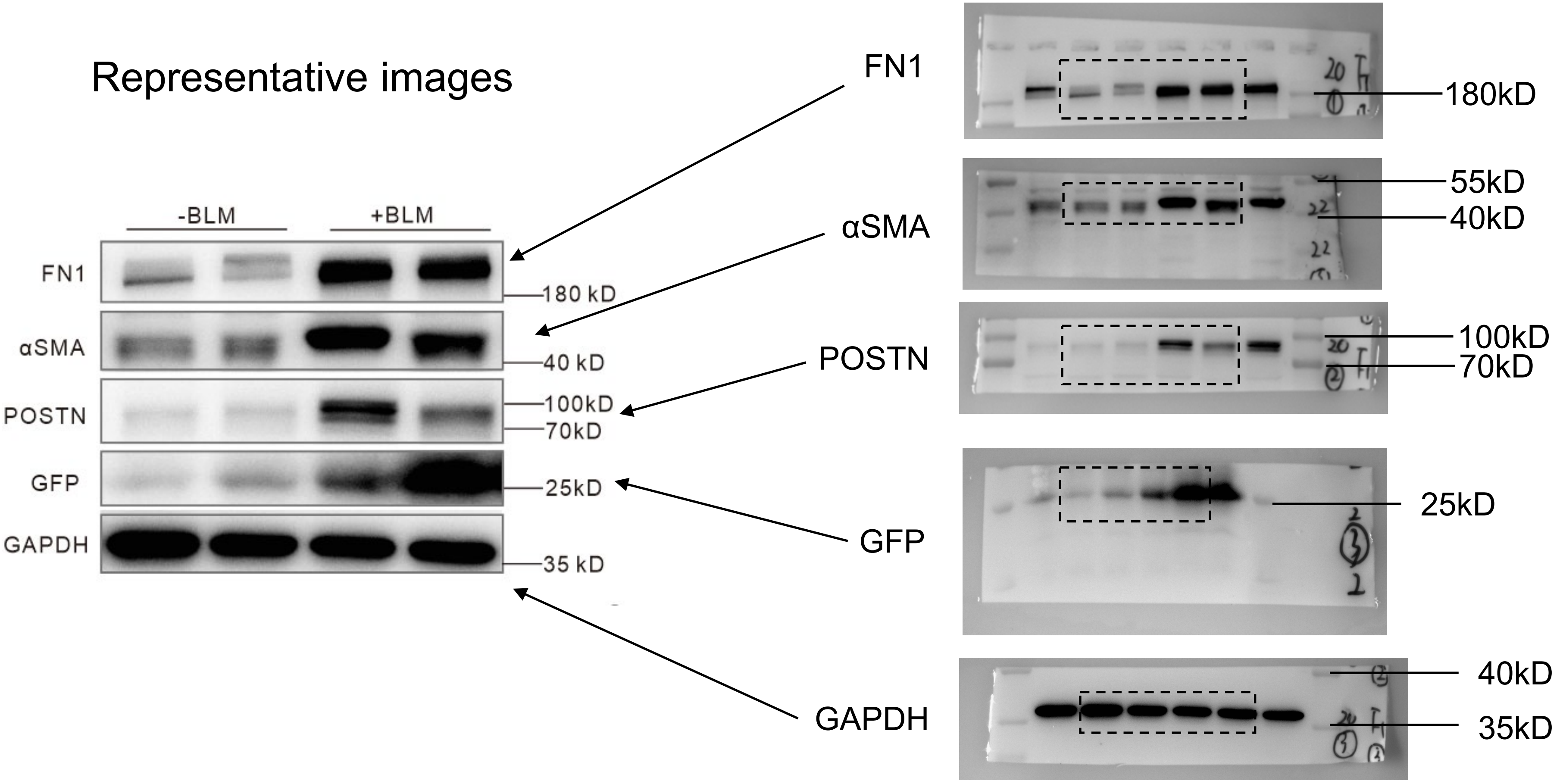

Full unedited blot/gel for Figure 4F

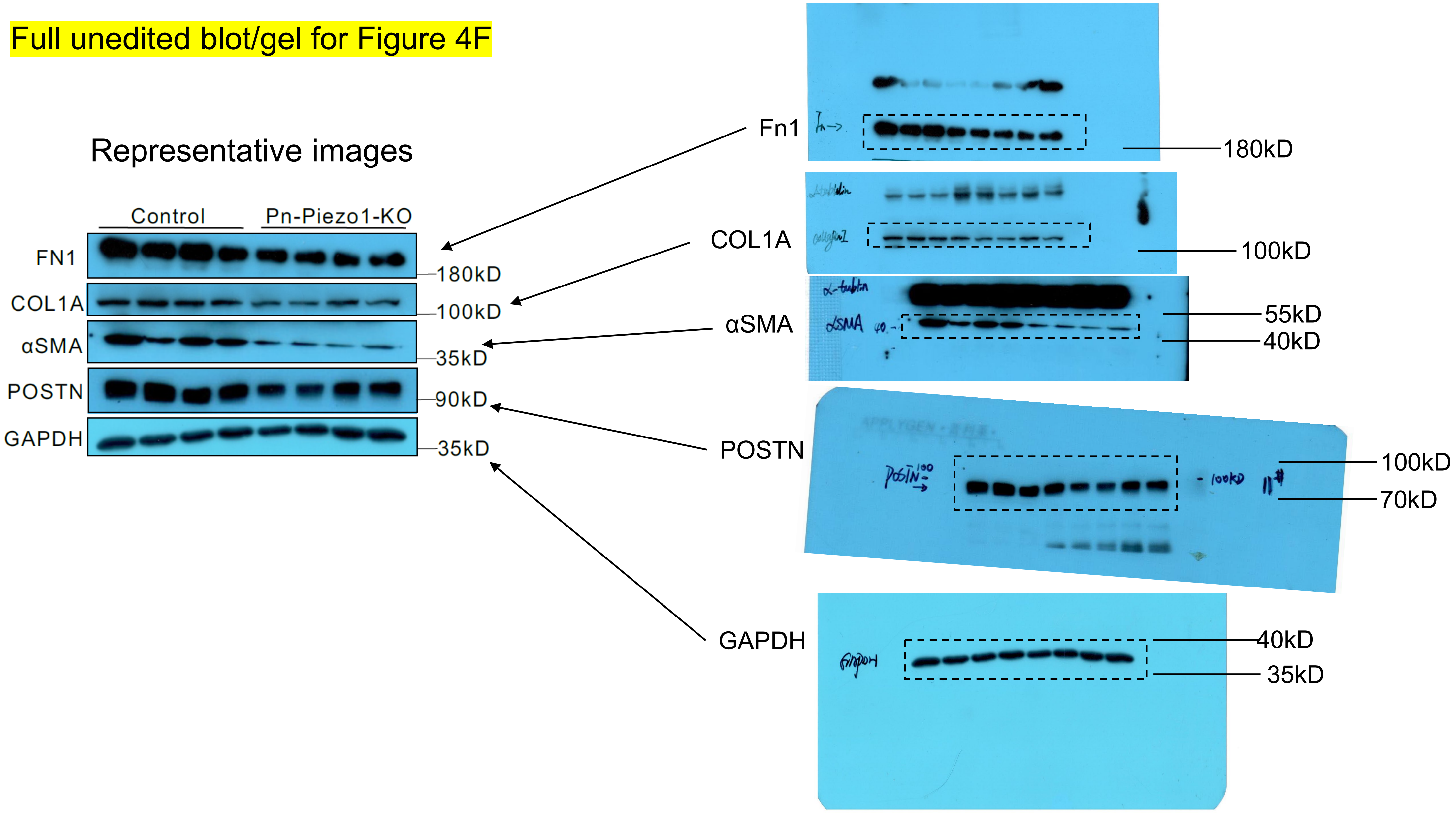

Full unedited blot/gel for Figure 7L

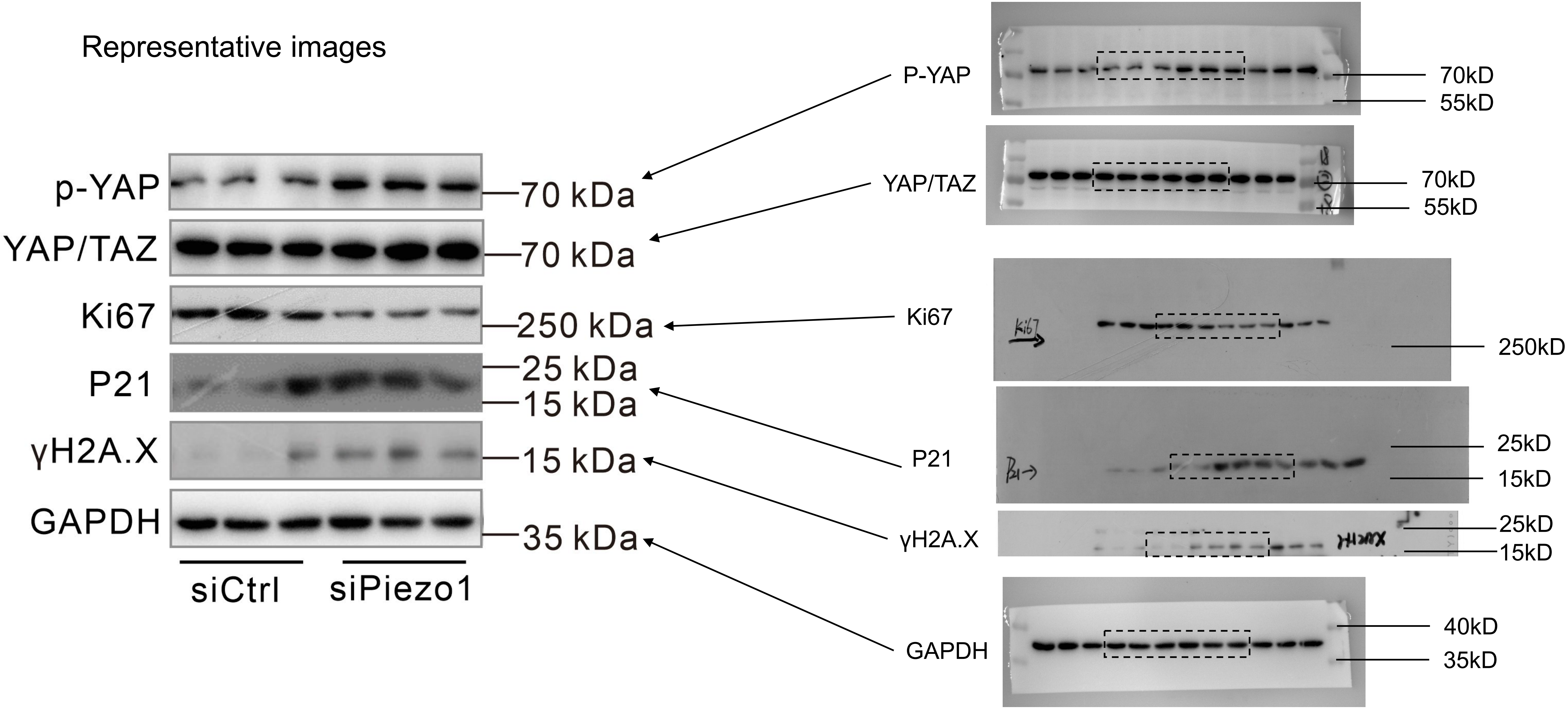

Full unedited blot/gel for Supplemental Figure 2E

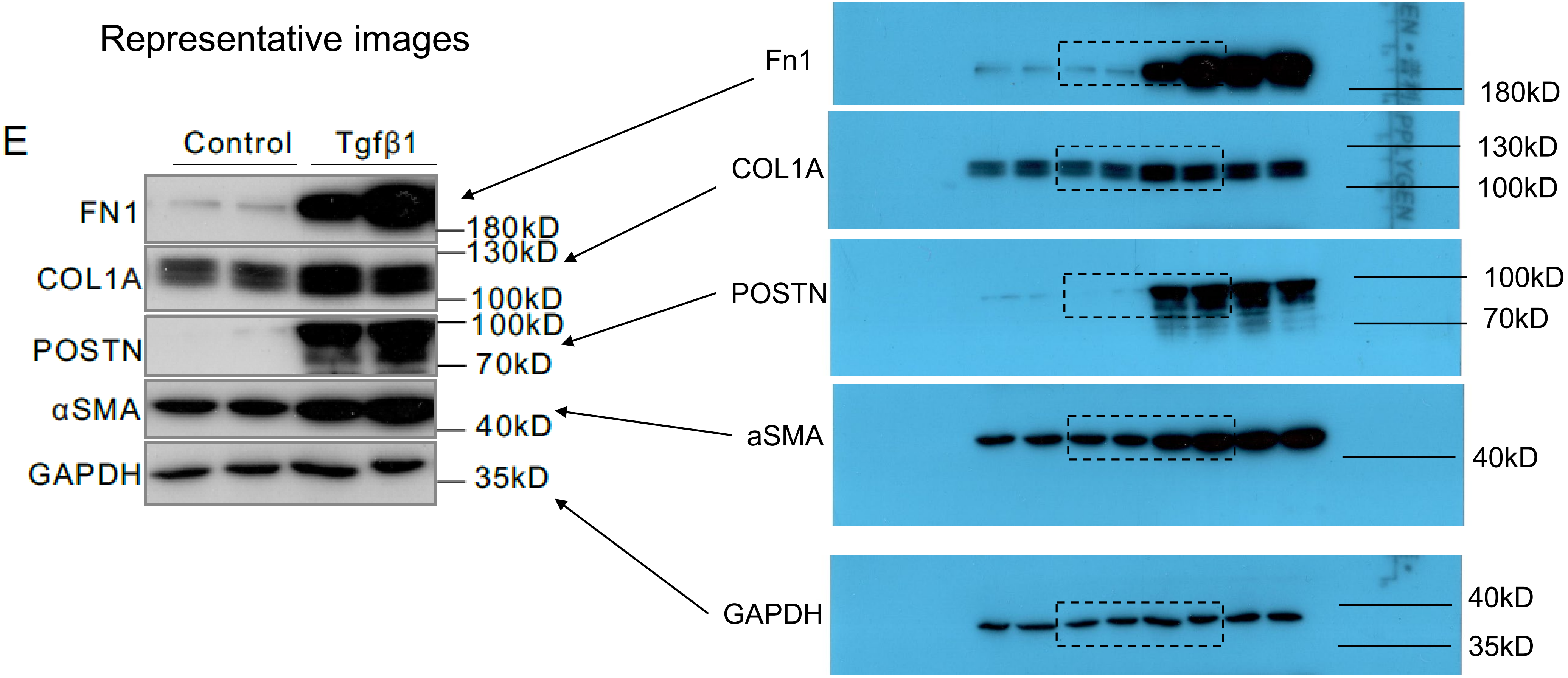

Full unedited blot/gel for Supplemental Figure 4D

Representative images

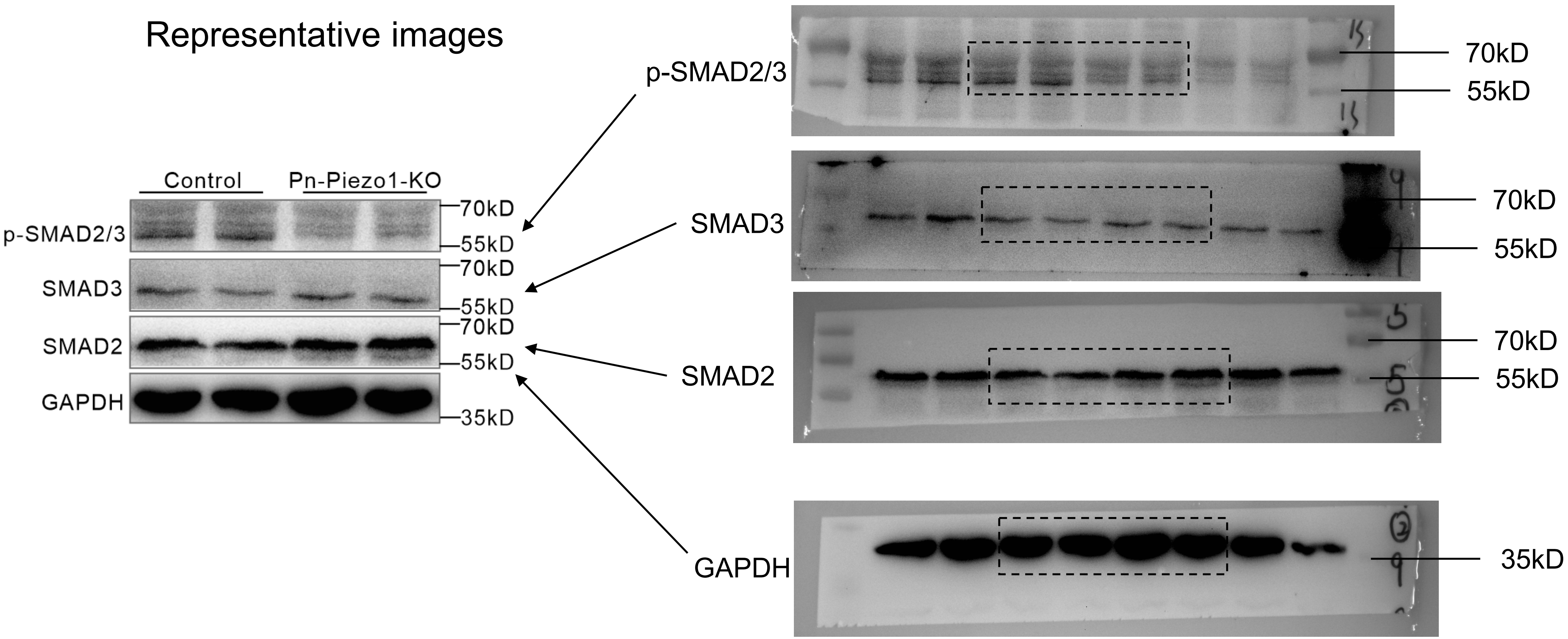

Full unedited blot/gel for Supplemental Figure 7E

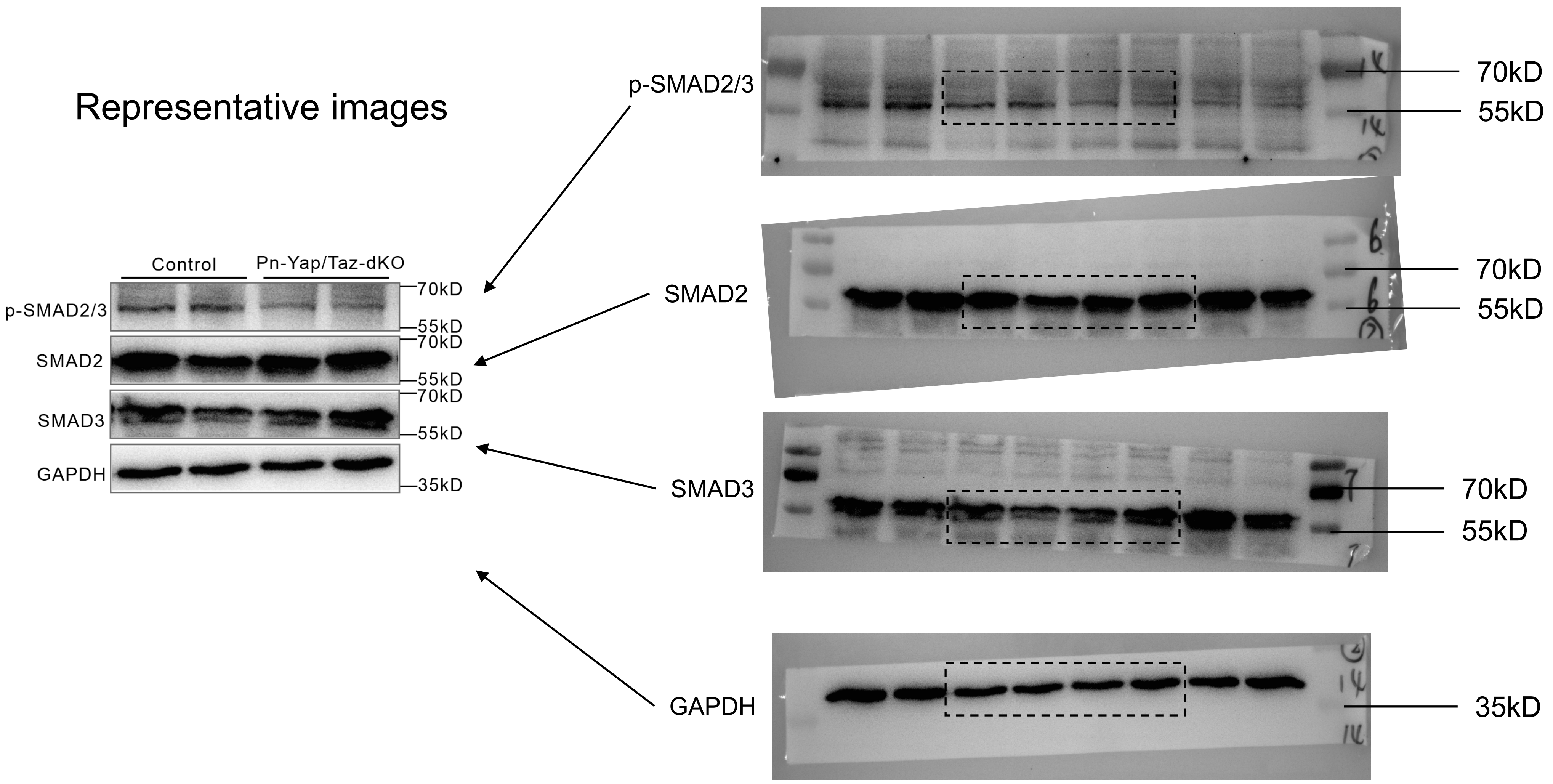

Full unedited blot/gel for Supplemental Figure 8A

Representative images

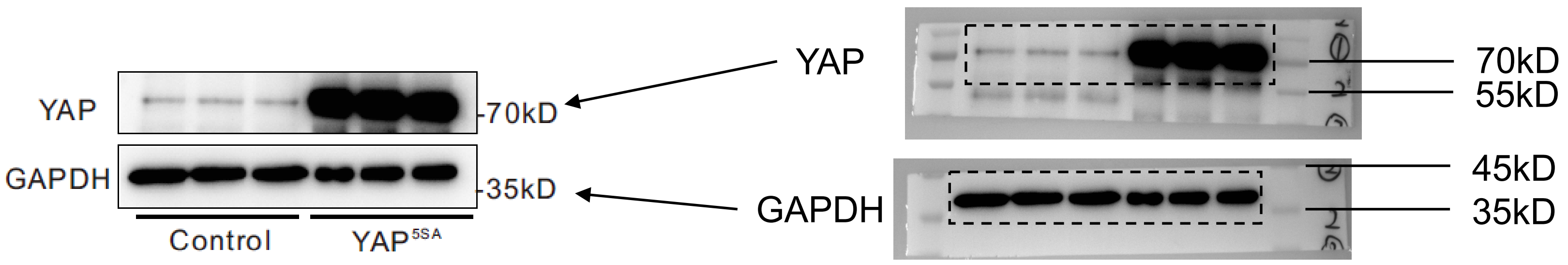

Full unedited blot/gel for Supplemental Figure 9J

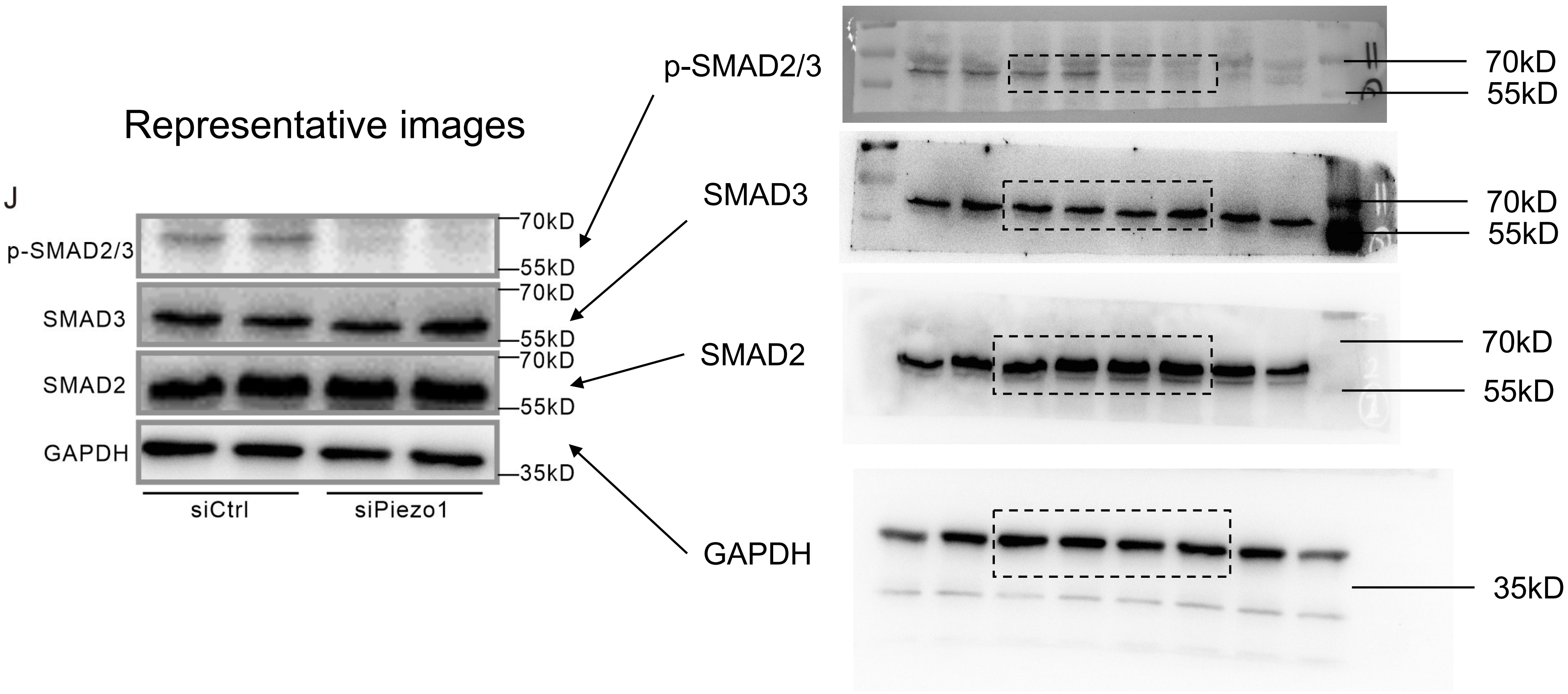

Supplement: Unedited blot and gel images [file jci-135-184158-s223.pdf]
